# Rio1 downregulates centromeric RNA levels to promote the timely assembly of structurally fit kinetochores

**DOI:** 10.1038/s41467-023-38920-9

**Published:** 2023-06-01

**Authors:** Ksenia Smurova, Michela Damizia, Carmela Irene, Stefania Stancari, Giovanna Berto, Giulia Perticari, Maria Giuseppina Iacovella, Ilaria D’Ambrosio, Maria Giubettini, Réginald Philippe, Chiara Baggio, Elisabetta Callegaro, Andrea Casagranda, Alessandro Corsini, Vincenzo Gentile Polese, Anna Ricci, Erik Dassi, Peter De Wulf

**Affiliations:** 1 Department of Cellular, Computational and Integrative Biology (CIBIO), University of Trento, Via Sommarive 9, 38123 Trento, Italy; 2 Department of Experimental Oncology, European Institute of Oncology, Via Adamello 16, 20139 Milano, Italy

**Keywords:** Kinetochores, Chromosome segregation, Centromeres, RNA decay

## Abstract

Kinetochores assemble on centromeres via histone H3 variant CENP-A and low levels of centromere transcripts (cenRNAs). The latter are ensured by the downregulation of RNA polymerase II (RNAPII) activity, and cenRNA turnover by the nuclear exosome. Using *S. cerevisiae*, we now add protein kinase Rio1 to this scheme. Yeast cenRNAs are produced either as short (median lengths of 231 nt) or long (4458 nt) transcripts, in a 1:1 ratio. Rio1 limits their production by reducing RNAPII accessibility and promotes cenRNA degradation by the 5’−3’exoribonuclease Rat1. Rio1 similarly curtails the concentrations of noncoding pericenRNAs. These exist as short transcripts (225 nt) at levels that are minimally two orders of magnitude higher than the cenRNAs. In yeast depleted of Rio1, cen- and pericenRNAs accumulate, CEN nucleosomes and kinetochores misform, causing chromosome instability. The latter phenotypes are also observed with human cells lacking orthologue RioK1, suggesting that CEN regulation by Rio1/RioK1 is evolutionary conserved.

## Abstract (web summary)

Kinetochores assemble on centromeres via histone H3 variant CENP-A and low levels of centromere transcripts (cenRNAs). Here the authors show the Rio1 kinase limits cenRNA production by reducing RNAPII accessibility and promotes cenRNA degradation by the 5’− 3’exoribonuclease Rat1.

## Introduction

Low levels of noncoding cenRNAs are required for faithful kinetochore assembly on centromeres (reviewed in^1–4^). This request is satisfied epigenetically (RNAi activity, the CEN histone code, CEN DNA and cenRNA nucleotide modifications^5–10^), by transcription factor Cbf1 (*Saccharomyces cerevisiae*)^11–13^ and CEN-binding protein CENP-B (humans)^14,15^, which curtail RNA polymerase (RNAPII) activity. In parallel, 3’−5’ nuclear exosome activity degrades excess cenRNAs^16–18^. Our understanding of cenRNAs and their activity(ies) remains limited since they exist in very low concentrations (0.002–0.03 molecules per *S. cerevisiae* cell^12,13^). This lack of knowledge is unfortunate, given that inordinate amounts of cenRNAs have been identified in malignancies^19–21^. Both elevated and reduced cenRNA levels caused chromosome instability^22,23^, a hallmark of cancer.

We previously reported that the essential and evolutionary conserved *S. cerevisiae* protein kinase/ATPase Rio1 downregulates RNA polymerase I (RNAPI) activity at the rDNA (nucleolus), while promoting *35S* pre-rRNA processing via the SSU processome. Both activities clear the rDNA, allowing for its condensation and segregation^24^. During that study, imaging and genetic interaction analyses placed Rio1 also at centromeres^25^, suggesting it might likewise regulate cenRNA production (by RNAPII) and processing. Given that Rio1 is conserved to humans (the oncogenic RioK1), we investigated this hypothesis in yeast and human cells.

At S-phase entry, *S. cerevisiae* kinetochores and centromeric nucleosomes disassemble, allowing the replisome and RNAPII to replicate and transcribe the centromeres, respectively. Within the following five minutes, the CEN nucleosomes reform and kinetochores reassemble onto them^26^. Excess cenRNAs become degraded before and/or after kinetochore recruitment. We noticed that Rio1 levels peaked at centromeres in early S-phase, indicating that the enzyme might contribute to one or more of the above activities. Indeed, depleting Rio1 led to enhanced local levels of RNAPII, elevated cenRNA concentrations, and untimely formed, structurally aberrant kinetochores. Using the Illumina NovaSeq 6000 RNA-sequencing platform we studied cen transcripts at physiological levels, and found that they are present in 2.0 ± 0.5 molecules per thousand cells (asynchronous culture), are produced largely from inside the pericentromere regions (few from within the centromeres), are heterogeneous in composition, cover the CEN core sequence (117 ± 2 bp) completely or partially, and exist as short (median length of 231 nt) or long transcripts (4458 nt). The latter all extend beyond the CEN core sequence. CenRNA levels differed significantly depending on the chromosomes they derived from. Some were produced from the upper strand, others from the lower strand. Depleting Rio1 caused a significant increase in the number of short cenRNA reads. In contrast, the absence of Rio1 activity did not affect the long cen transcripts as for processing (length) and production, suggesting that the short cenRNAs derive from enhanced de novo transcription upon Rio1 depletion. This occurrence is supported by the number of short cenRNA read start sites that strongly increased following Rio1 removal. Beside the CEN-containing RNAs, we also identified transcripts that derived from the flanking pericentromere sequences. These included both coding and noncoding transcripts but depleting Rio1 only affected the noncoding pericenRNAs, which existed on average at one transcript per cell (thus at more than two orders of magnitude higher than the cenRNAs).

We demonstrate that Rio1 restricts CEN and periCEN chromatin access for RNAPII. The cen- and noncoding pericenRNA levels are reduced by the RNA degrading 5’−3’ exoribonuclease Rat1, in parallel to the 3’−5’ nuclear exosome. Together with repressive transcription factor Cbf1^11^, histone H2.A variant Htz1^12,27^, and the assembled kinetochore itself, Rio1 establishes the centromere as a highly negative environment for cenRNA production. Depleting human orthologue RioK1 similarly caused cenRNA accumulation and faulty CEN nucleosome and kinetochore formation, culminating in chromosome instability, suggesting that CEN regulation by Rio1/RioK1 is evolutionary conserved.

## Results

### Characterisation of budding yeast cen- and pericen transcripts

**Fig. 1:**
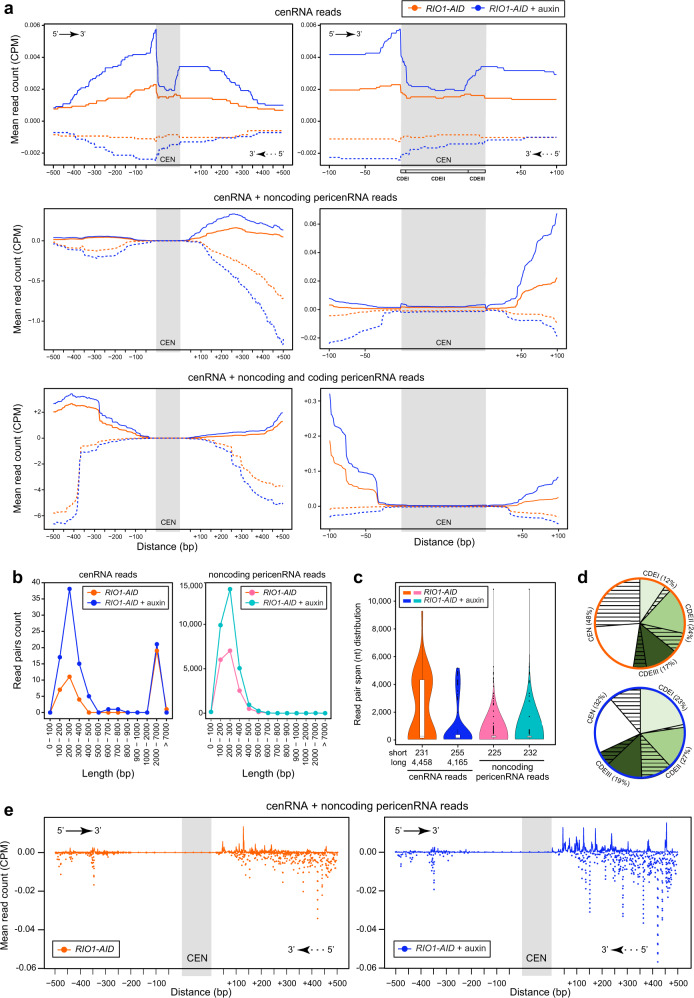
Characterisation of centromere and pericentromere transcripts. **a** Upper, middle and bottom plots: the number of cenRNA reads; cen- and noncoding pericenRNA reads; cen-, noncoding pericen-, and coding pericenRNA reads (as mean read counts), respectively, deriving from all sixteen chromosomes and tallied from three independent experiments (*n* = 3), are plotted against the sequences from which they derived. The CEN core regions are indicated by a grey column. The full lines represent the read pairs deriving from the upper strand, the dashed lines those deriving from the lower strand. Orange lines: *RIO1-AID* cells treated with a mock for 1 h (*n* = 3), blue lines: *RIO1-AID* cells treated with 500 µM auxin for 1 h (*n* = 3). The left and right plots comprise 500 bp and 100 bp of the periCEN regions surrounding the CEN core sequences, respectively. **b** Left plot: length-based distribution of the cenRNA reads deriving from all chromosomes. Right plot: length-based distribution of all noncoding pericenRNA reads deriving from all chromosomes. The cen- and noncoding pericenRNA reads identified in *RIO1-AID* cells treated with a mock (tallied from three replicate experiments, *n* = 3) are indicated in orange and pink, respectively. The cen- and noncoding pericenRNA reads identified in *RIO1-AID* cells (tallied from three replicate experiments, *n* = 3) treated with auxin are shown in dark and light blue, respectively. **c** Violin plot of the length-based distribution of cenRNA reads (classified by short or long) and of noncoding pericenRNA reads in *RIO1-AID* cells treated with a mock or auxin (three replicate experiments, *n* = 3). **d** Distribution of cenRNA reads that contain the entire CEN core sequence (“CEN”) or that terminate in a specific CEN element (CDEI, CDEII, CDEIII) in *RIO1-AID* cells treated with a mock (encircled in orange) or auxin (encircled in blue) (three experimental replicates, *n* = 3). The filled and open slices indicate the percentage of reads deriving from the upper and lower strand, respectively. **e** Distribution of the start sites of cen- and noncoding pericenRNA reads in *RIO1-AID* cells treated with a mock (left plot) or auxin (right plot). Full and dashed lines: read start sites localising on the upper and lower strand, respectively (three experimental replicates, *n* = 3).

To determine whether Rio1 manages cenRNA levels in *S. cerevisiae*, similar to it downregulating *35S* pre-rRNA production and promoting their subsequent processing^24^, we isolated the RNAs from three mid-exponential cultures of a *RIO1-AID* degron strain treated for 1 h with a mock or 500 µM auxin (to deplete Rio1). Next, all rRNAs were removed to expose the less abundant transcripts. Specific transcript enrichments (centromeric or other) were not performed. All RNAs were then converted into cDNAs and deep-sequenced in 100nt paired-end mode. The reads corresponding to centromere transcripts (arbitrarily defined as read pairs containing the 117 ± 2 bp CEN core sequence or a part of it) deriving from all sixteen chromosomes in the three yeast cultures were tallied and plotted against the genome region (CEN core ±500 bp) they originated from (upper two plots in Fig. 1a). This was done for both treatment conditions (the cenRNA read pairs deriving from the individual chromosomes and tallied from the three replicates are plotted in Supplementary Fig. 1). The orange and blue lines represent the sum of read pairs deriving from three *RIO1-AID* cultures treated either with a mock or auxin (Supplementary Fig. 2d), respectively. The number of cenRNA reads counted in the mock-treated cultures (42 read pairs) increased 2.3-fold in the Rio1-depleted cells (98 read pairs) (Supplementary Fig. 2a). The full and dashed lines represent the read pairs deriving from the upper or lower strand, respectively. As evidenced by these profiles, most cenRNA reads originated from the plus strand (10/16 chromosomes). Nine randomly selected, computationally identified cenRNA read pairs were validated by PCR-based analysis of cDNAs (Supplementary Fig. 2b).

The cenRNA read numbers varied significantly between the chromosomes (Supplementary Fig. 2c), possibly due to the local chromatin context affecting RNAPII access and activity (e.g., nucleosome-rich and compacted chromatin versus nucleosome-poor/free and open chromatin)^11–13,28^. In any case, their overall levels were extremely low, corresponding to 2.0 ± 0.5 molecules per 1000 cells, using the number of mRNAs/cell produced from *DOA1*, *KAP104*, *POL1*, and *PDR5* (2.6, 5.0, 3.1, and 13.4, respectively) as the references^29^, confirming previous estimates^12,13^. The cenRNA read pairs were very heterogeneous with respect to their length^8–10^. In the cultures treated with a mock, they existed -in equal amounts- as short (<1000 nt) or long (>1000 nt) reads (same areas underneath the orange peaks in Fig. 1b). The short cenRNA reads had a median length of 231 nt (Fig. 1c). The shortest cen read pair identified was 125 nt long and derived from chromosome 6. The long cenRNA reads had a median length of 4458 nts (Fig. 1c), with the longest one comprising 9376 nt and deriving from chromosome 1. The cenRNA reads were also heterogeneous with regard to their composition; most (79%) initiated in the flanking pericentromeres, with the rest originating from within the CEN core and extending beyond. As shown in Fig. 1d (upper pie chart), 48% comprised the entire CEN sequence, whereas the others initiated or terminated in one of the three centromere elements (CDEI-III). The filled slices represent the percentage of read pairs deriving from the upper strand, the open slices those deriving from the lower strand.

**Fig. 2:**
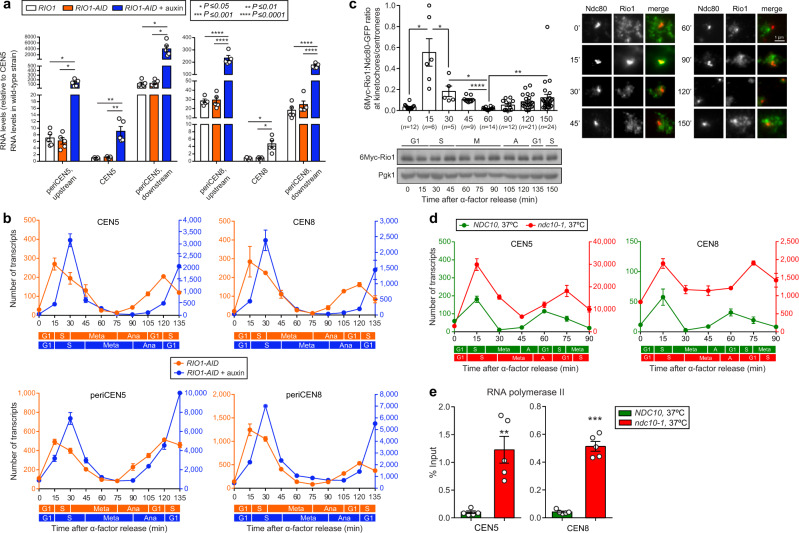
Cell cycle stage-dependent regulation of cen- and noncoding pericenRNA levels. **a** Cen- and noncoding pericenRNA levels deriving from chromosomes 5 and 8 as measured (RT-qPCR) in *RIO1* cells, and in *RIO1-AID* cells treated with a mock or 500 µM auxin. Their concentrations were normalised to those of *ACT1* mRNA (RT-qPCR), and referenced to the corresponding transcript levels in the *RIO1* strain (value = 1). The singular data (white circles) derived from five biological experiments (*n* = 5) and are shown combined as mean ± SEM (standard error of the mean). Confidence levels (*P*-values) were calculated with the unpaired, two-tailed Student’s *t* test. **b** Cen5 and noncoding pericen5 RNAs, cen8 and noncoding pericen8 RNAs measured by RT-qPCR through a synchronous cell cycle (α-factor arrest-and-release) in the *RIO1-AID* strain treated with a mock or 500 µM auxin (three replicates per condition, *n* = 3). Transcript levels were normalised to those of *ACT1*. *P*-values (measured using the unpaired, two-tailed Student’s *t* test) comparing the mock and auxin data are shown in Supplementary Fig. 3e. **c** Plot: immunofluorescence imaging (IF)-based analysis of 6Myc-Rio1 (anti-Myc) and Ndc80-GFP (anti-GFP) levels in spread nuclei isolated from yeast through the cell cycle (α-factor arrest-and-release). *n* indicates the number of cells (circles) analysed, deriving from three replicate experiments. The combined data are shown as mean ± SD (standard deviation). Blots: western blots of 6Myc-Rio1 (anti-Myc) and Pgk1 (anti-Pgk1, loading control) through the cell cycle (from the cell samples taken during the upper-plot experiment). Images: representative IF images of spreads analysed at the indicated time points. **d** Cen5 and cen8 RNA numbers measured in wild-type yeast (*NDC10*) and in an *ndc10-1* mutant synchronously released into the cell cycle at 37 °C. Transcript levels were normalised to those of *ACT1*. *P*-values (unpaired, two-tailed Student’s *t* test) comparing the mock and auxin data are shown in Supplementary Fig. 3f. **e** Relative RNA polymerase II levels at CEN5 and CEN8 as determined by ChIP-qPCR analysis in asynchronous *NDC10* and *ndc10-1* cultures following a 3 h shift from 25 °C to 37 °C. The error bars represent SEM. *P*-values were calculated with the unpaired, two-tailed Student’s *t* test.

Upon Rio1 depletion, the number of cenRNA read pairs significantly increased, with the degree of increase again depending on the chromosome from which they derived (Supplementary Fig. 2c). This increase was also validated by RT-qPCR analyses (Fig. 2a, Supplementary Fig. 3a–c). Interestingly, only the number of short cenRNA reads rose following Rio1 depletion, whereas the amount of long cenRNA reads did not, resulting in a short-to-long cenRNA ratio of 3:1 (Fig. 1b). The new short cenRNA reads derived from de novo synthesis rather than from long cenRNA processing as suggested by the surge in the number of read start sites following Rio1 depletion (Fig. 1e). While the percentage of cenRNA reads initiating from within the pericentromeres remained the same following Rio1 removal, less cenRNA reads comprised the entire CEN sequence (32%), as more cenRNA read pairs terminated inside the CEN core sequence, especially within the CDEI element (Fig. 1d, lower pie chart) to which transcription factor Cbf1 binds^11^.

Besides cenRNA read pairs, we also distinguished pericentromeric RNA read pairs, arbitrarily defined as reads covering completely or partially the 500 bps that flank up- or downstream the CEN core but end at the centromere border (middle and lower plots in Fig. 1a; Supplementary Fig. 1). The pericenRNA reads were heterogeneous in length and composition, and comprised both noncoding reads (median length of 225 nt, Fig. 1c) and coding (mRNA) read pairs, which derived from the 22 ORFs residing entirely or partially within the 500 bp periCEN regions across the genome. Of note, no pericen mRNAs were identified within 200 bp surrounding the CEN cores. All noncoding pericenRNA reads tallied from the mock-treated cultures amounted to 16,369 read pairs, thus exceeding the cenRNA reads 390-fold, and abiding in an average of 1.0 (± 0.2) molecule per cell (Fig. 1a–c, e and 2a; Supplementary Fig. 1, 2b and 3a–c). The coding pericenRNA read pairs exceeded the noncoding pericenRNA reads on average 10-fold (Fig. 1a, Supplementary Fig. 1). Again, the cenRNA-to-pericenRNA read pair ratios varied between the chromosomes from which they derived (Supplementary Fig. 2e). Depleting Rio1 led to an accumulation of only the noncoding pericenRNA reads (1.9-fold increase from 16,369 reads to 30,346 reads), establishing the noncoding pericenRNA-to-cenRNA read pair ratio at 310 in the absence of Rio1. Their build up following Rio1 removal was confirmed by RT-qPCR analysis (Fig. 2a, Supplementary Fig. 3a–c). Similar to the short cenRNA read pairs, the measured increase in noncoding pericenRNA reads was due to de novo synthesis from newly emerged read starts sites (Fig. 1e). Henceforth, only the noncoding pericen transcripts will be examined and measured by RT-qPCR analysis (using oligomer pairs localising within 200 bp from the CEN border) as their levels depend on Rio1 activity, but will next be indicated for reasons of simplicity as “pericenRNAs/transcripts”.

### Rio1 downregulates cen- and pericenRNA levels in early S-phase

RT-qPCR based analysis of cen- and pericenRNA levels from G1 through a synchronous cell cycle (Fig. 2b, Supplementary Fig. 3d, e) revealed that the levels of both RNAs peaked in early S-phase, when the CEN and periCEN regions replicate. As expected, the pericen transcripts existed at much higher levels than the cenRNAs. The cen and pericen transcript concentrations next decreased through S-phase. In metaphase, cenRNAs became largely undetectable while pericen transcripts were identified at low levels. At anaphase onset, the numbers for both started to rise, to then peak again in S-phase. In yeast depleted of Rio1, which is characterised by a 15 min S-phase delay due to retarded rDNA replication^24^, the cell cycle profiles of the cen- and pericenRNAs were maintained, albeit at levels (quantified by RT-qPCR) that were 10- to 20-fold higher than those measured in the mock-treated cells (Fig. 2b, Supplementary Fig. 3d, e). To probe whether the presence of Rio1 at centromeres correlated with the above RNA profiles, we tracked the kinase by immunofluorescence (IF) imaging from G1 through the cell cycle (kinetochore protein Ndc80 as the internal reference) (Fig. 2c). A dynamic localisation pattern emerged that matched those of the cen- and pericenRNAs, suggesting a functional relationship between them. Since the protein levels of Rio1 remained stable through the cell cycle (Fig. 2c), nuclear import and export could explain its dynamics at centromeres/kinetochores given that Rio1 accumulated in the nucleus of nuclear export mutant *crm1-1*^30^.

To determine whether kinetochore occupancy restrains RNAPII activity at centromeres, we enriched both wild-type (*NDC10*) yeast and the temperature-sensitive *ndc10-1* mutant with α-factor in G1 at room temperature, and then released the cells in growth medium that was preheated at 37 °C. At this temperature, Ndc10-1 becomes inactivated and unable to initiate kinetochore recruitment^31^. We observed the typical cell cycle profile for cenRNAs albeit at levels that were 20- to 175-fold higher (depending on the centromere) than those measured in wild-type yeast (Fig. 2d, Supplementary Fig. 3f). Next, anti-RNAPII chromatin immunoprecipitation followed by qPCR analysis (ChIP-qPCR) revealed a significant increase in RNAPII occupancy at centromeres in the *ndc10-1* mutant at 37 °C (Fig. 2e) indicating that assembled kinetochores reduce RNAPII activity, likely contributing to the drop in cenRNA levels following kinetochore reformation in early S-phase (Fig. 2b).

### Rio1 downregulates RNAPII access and promotes cen- and pericenRNA turnover

**Fig. 3:**
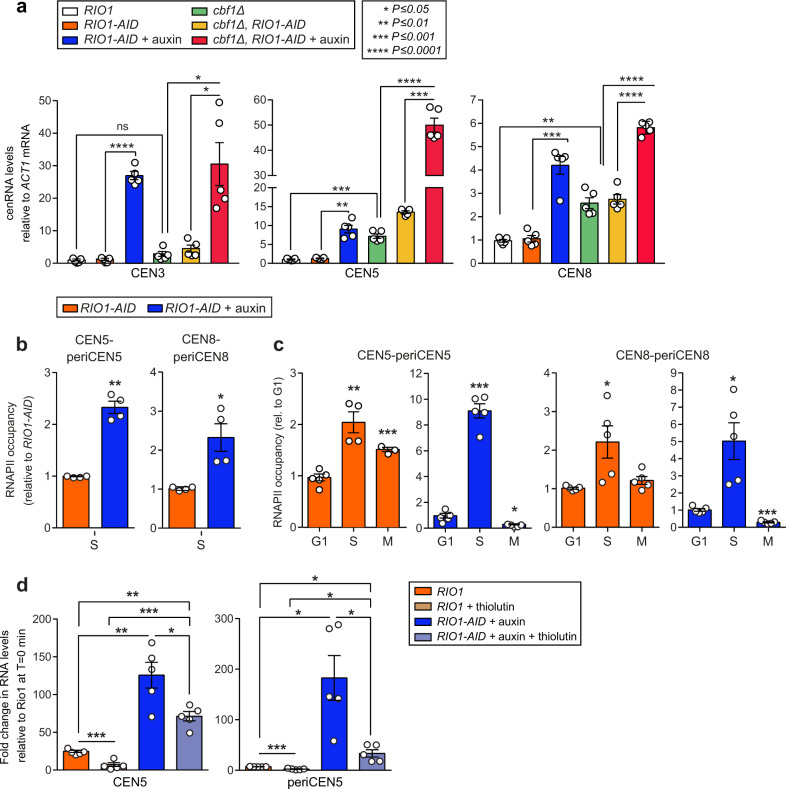
Rio1 downregulates RNAPII recruitment and activity at (peri)centromeres. **a** CenRNA levels measured by RT-qPCR analysis in wild-type yeast (*RIO1*), in the *RIO1-AID* strain treated with 500 µM auxin or a mock, in a *cbf1*∆ mutant, and in a *cbf1∆ RIO1-AID* strain treated with 500 µM auxin or a mock. Transcript concentrations were normalised to those of *ACT1* and then referenced to the corresponding cenRNA levels measured in the wild-type strain (value = 1). Singular data (white circles) deriving from five biological replicates (*n* = 5) are shown combined as mean ± SEM. *P*-values were calculated with the unpaired, two-tailed Student’s *t* test. **b** RNA polymerase II levels at CEN5-periCEN5 and CEN8-periCEN8 as determined by anti-RNAPII ChIP-qPCR analysis in early S-phase *RIO1-AID* cells (following α-factor arrest-and-release) in the presence of 500 µM auxin or a mock. The data were normalised to those measured in the mock-treated cells (four replicate experiments (*n* = 4), shown as mean ± SEM). *P*-values were calculated with the unpaired, two-tailed Student’s *t* test. **c** RNA polymerase II levels at CEN5-periCEN5 and CEN8-periCEN8, measured by anti-RNAPII ChIP-qPCR analysis in G1, S-phase, and metaphase *RIO1-AID* cells, following α-factor arrest-and-release in the presence of 500 µM auxin or a mock. The data were normalised to those measured in G1 (α-factor arrested cells). The results derived from five replicate experiments (*n* = 5) and are shown as mean ± SEM. *P*-values were calculated with the unpaired, two-tailed Student’s *t* test. **d** Cen5 and pericen5 RNA levels measured by RT-qPCR analysis in *RIO1* and *RIO1-AID* cells treated with a mock, 3 µM thiolutin and/or 500 µM auxin. The cells were released from G1 and analysed in early S-phase (five experimental replicates per condition, *n* = 5). Transcript levels were normalised to those measured in the untreated wild-type strain at T = 0 min. The singular data (white circles) are shown combined as mean ± SEM. *P*-values were calculated with the unpaired, two-tailed Student’s *t* test.

Transcription factor Cbf1 has been reported to either activate^32^ or repress^11–13^ CEN transcription. Compared to wild-type yeast, we found that the *cbf1Δ* mutant contained 2.5-7.5-fold higher cenRNA levels (depending on the centromere analysed; Fig. 3a), indicating that Cbf1 restrains CEN transcription, likely by acting as a roadblock for RNAPII^11^. Noteworthy, deleting Cbf1 had an 8-fold lower effect on cenRNA accumulation than depleting Rio1 (Fig. 3a). Eliminating both proteins (*cbf1Δ RIO1-AID* strain +auxin for 1 h) triggered an additive increase in cenRNA numbers (Fig. 3a), suggesting that Cbf1 and Rio1 independently curtail cenRNA levels.

To determine whether Rio1 restrains RNAPII recruitment to the CEN and periCEN sequences, as suggested by new transcript read start sites emerging locally following Rio1 depletion (Fig. 1e), we quantitated RNAPII occupancy at the read start sites-enriched CEN-periCEN border regions. Specifically, anti-RNAPII ChIP-qPCR analyses were performed on early S-phase *RIO1-AID* cells released from G1 in the presence of a mock or 500 µM auxin. Compared to the mock-treated cells, the local RNAPII levels more than doubled in the cells depleted of Rio1 (Fig. 3b). Next, we tracked RNAPII levels in *RIO1-AID* cells released from G1 through S-phase and into metaphase in the presence of auxin or a mock. As expected, RNAPII levels peaked in S-phase, but the enzyme was also identified at low levels in G1 and metaphase (Fig. 3c), suggesting transcription activity occurring also at those cell cycle stages, a hypothesis that is compatible with the low concentrations of cen and pericen transcripts measured in G1 and metaphase (Fig. 2b). To further validate the involvement of Rio1 in regulating RNAPII transcription activity, we released wild-type (*RIO1*) and *RIO1-AID* cells from G1 into early S-phase in the presence of auxin (or a mock) and RNAPII inhibitor thiolutin. Thiolutin treatment (30 min during the G1-to-S-phase transition) reduced cen- and pericenRNA levels in both strains, as expected (Fig. 3d). However, the cen and pericen transcript concentrations measured in the RNAPII-inhibited, Rio1-depleted cells well exceeded those measured in the thiolutin-treated wild-type cells. These observations imply that removing Rio1 derepressed RNAPII activity while stabilising the produced cen- and pericenRNAs (Fig. 3d).

### Rio1 promotes cen- and pericenRNA turnover by Rat1

**Fig. 4:**
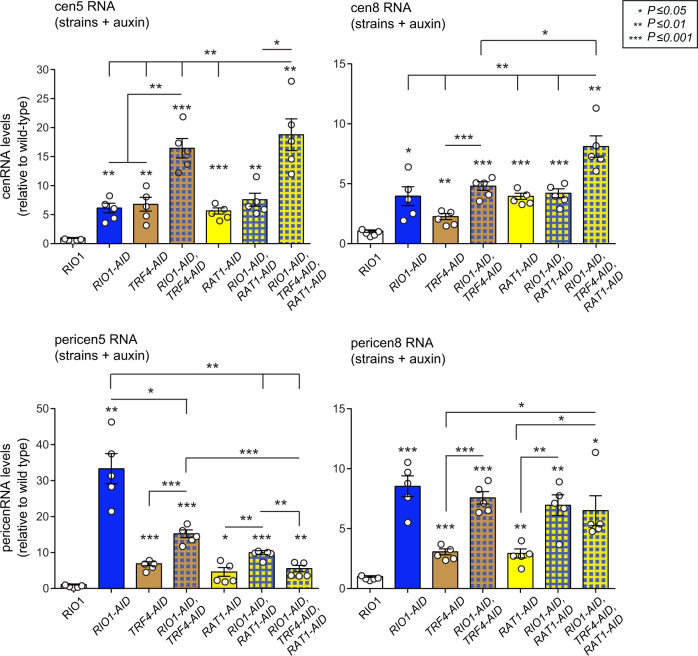
Regulation of cen- and pericenRNA levels by Rio1, Rat1, and TRAMP/nuclear exosome activities. Cen- and pericenRNA levels measured in the wild-type (*RIO1*), *RIO1-AID, TRF4-AID, RIO1-AID TRF4-AID, RAT1-AID*, *RIO1-AID RAT1-AID*, and *RIO1-AID TRF4-AID RAT1-AID* strains treated for 1 h with 500 µM auxin (five experimental replicates for each strain (*n* = 5)). The indicated cen- and pericenRNAs (upper and lower plots, respectively) were quantitated by RT-qPCR analysis, normalised to those of *ACT1* mRNA (RT-qPCR), and then referenced to the values determined for the *RIO1* strain (value = 1). The striped-line patterns indicate double and triple protein depletions, with each color representing one depleted protein. The singular data (white circles) are shown combined as mean ± SEM. *P*-values were calculated with the unpaired, two-tailed Student’s *t* test.

The 3’−5’ nuclear exosome, supported by the RNA helicase TRAMP (Trf4/Air2/Mtr4 Polyadenylation) complex, surveils and degrades cenRNAs in *S. cerevisiae*^12,16^. Inactivating TRAMP activity (*TRF4-AID* strain +auxin for 1 h, Supplementary Fig. 4a), which prohibits nuclear exosome activity, led to a significant increase in both cen and pericen transcripts. Depleting both Rio1 and Trf4 (*RIO1-AID TRF4-AID* strain +auxin for 1 h) led to an additive accumulation of cen- and pericenRNAs, suggesting that Rio1 and the nuclear exosome promote cen and pericen transcript turnover in parallel fashion (Fig. 4, Supplementary Fig. 4b, c).

Given that Rio1^24^, 3’−5’ TRAMP/nuclear exosome, and 5’−3’ exoribonuclease Rat1^21^ activities promote pre-rRNA processing and degradation, we next probed the involvement of RNase Rat1 in cen and pericen transcript turnover. Depleting Rat1 (*RAT1-AID* strain +auxin for 1 h, Supplementary Fig. 4a) caused a strong increase in cen- and pericenRNA levels (Fig. 4, Supplementary Fig. 4b, c). Removing both Rio1 and Rat1 (*RIO1-AID RAT1-AID* strain +auxin for 1 h) did not trigger an additive buildup of cenRNAs, suggesting that both enzymes act in one pathway. Depleting all three protein activities (*RIO1-AID TRF4-AID RAT1-AID* strain +auxin for 1 h) caused an increase in cenRNA levels, indicating that they become degraded in parallel fashion by Rio1-Rat1 and the nuclear exosome (Fig. 4, Supplementary Fig. 4b, c). As for the pericenRNA levels; when Rat1, Rio1, and Trf4 were removed in double or triple combinations, complex outcomes were observed. Indeed, while the pericen transcript levels increased, they never exceeded those measured in the Rio1-depleted cells (Fig. 4, Supplementary Fig. 4b, c). Possibly, the already high pericenRNA concentrations accumulating following Rio1 removal could reach detrimental levels when combined with Trf4 and/or Rat1 depletions; triggering activities that may antagonise excess pericenRNA buildup.

Next, three affinity purifications of Rio1 followed by mass spectrometry analysis revealed strong interactions with proteins that regulate RNAPII activity and process RNAs (Supplementary Data 1). These data extended our previous yeast two-hybrid (Y2H) screens with Rio1^25^. However, Rio1 did not co-purify Rat1 (the Y2H screens also did not identify Rat1 as an interactor of Rio1^25^). Reversely, three purifications of Rat1 also did not reveal Rio1, suggesting that both collaborate through a(n) intermediate(s). Twenty-six proteins co-purified with both Rio1 and Rat1 (Supplementary Fig. 5, Supplementary Data 1) including Rat1 regulators Las1 and Grc3^33^, which could functionally connect Rio1 and Rat1.

### Rio1 promotes timely and correct kinetochore formation

**Fig. 5:**
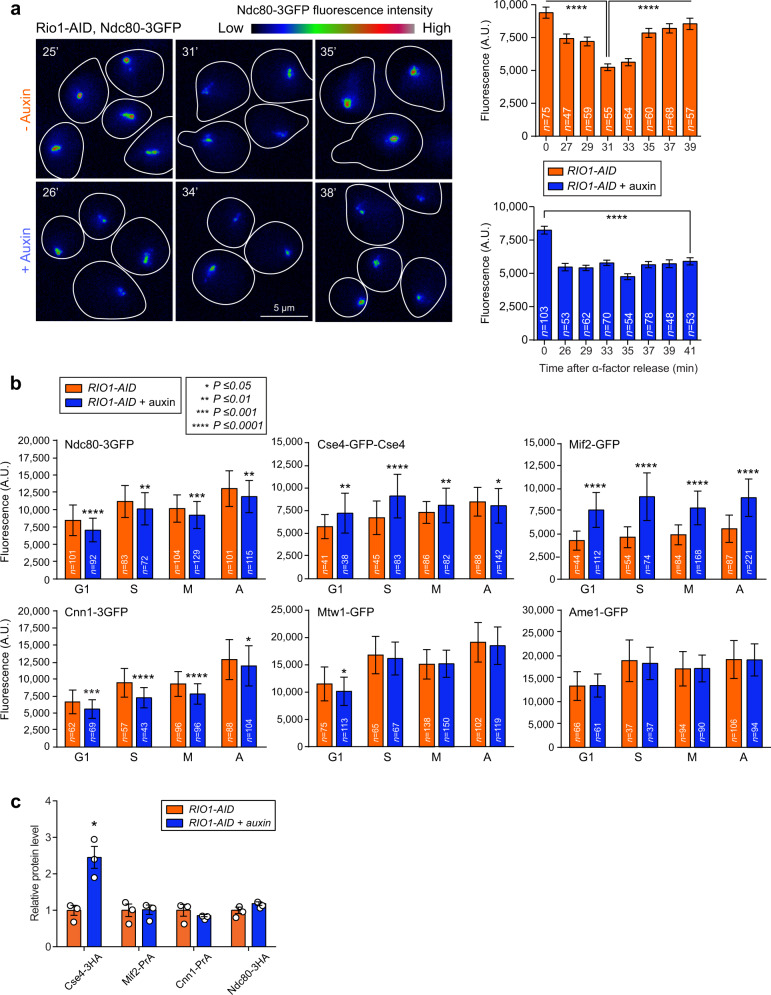
Rio1 promotes the timely formation of structurally fit kinetochores. **a** Real-time quantitative analysis of Ndc80-3GFP fluorescence levels at kinetochores in a *RIO1-AID* strain released from G1 in the presence of 500 µM auxin or a mock, and tracked through S-phase. The number of cells analysed (*n*) at each time-point derived from three independent biological experiments. The cells were imaged across different microscopy fields through 20–30 Z-planes, which were then vertically projected at maximum intensity to measure the fluorescence signals. A.U. arbitrary units. The data are plotted as mean ± SD, *P*-values were calculated with the unpaired, two-tailed Student’s *t* test. **b** Fluorescence levels of GFP-labelled kinetochore proteins at spindle-bound kinetochores in *RIO1-AID* strains treated with 500 µM auxin or a mock. The number of cells analysed (*n*) per cell cycle stage (G1, S-phase (S), metaphase (M), and anaphase (A)) derived from three independent biological experiments. The cells were imaged across different microscopy fields through 20–30 Z-planes, which were then vertically projected at maximum fluorescence intensity for signal measurement. A.U. arbitrary units. The data are plotted as mean ± SD. *P*-values were calculated with the unpaired, two-tailed Student’s *t* test. **c** Whole-cell levels of epitope-tagged Cse4, Mif2, Cnn1, and Ndc80 measured by western blot in *RIO1-AID* cells treated with auxin or a mock. OsTir1 or Pgk1 acted as the loading controls and internal references for relative quantifications (blots are shown in Supplementary Fig. 7a). The measurements were normalised to those quantitated in the mock-treated cells (value = 1). The singular data (white circles) derived from three independent biological experiments (*n* = 3) and are combined as mean ± SEM. *P*-values were calculated with the unpaired, two-tailed Student’s *t* test.

To determine whether Rio1 depletion, and the consequent accumulation of cen- and pericenRNAs, influences kinetochore assembly, we tracked kinetochore protein Ndc80-3GFP in live *RIO1-AID* cells from G1 through S-phase. Images were acquired every two minutes (Fig. 5a). Upon treatment with a mock, Ndc80 levels decreased synchronously across all centromeres in early S-phase, consistent with kinetochores disassembling. Within five minutes, Ndc80 signals at kinetochores increased, denoting kinetochore re-formation^26^. In the auxin-treated cells, kinetochore dis- and re-assembly were untimely (asynchronous) and the Ndc80 levels at kinetochores were also lower, suggesting aberrant kinetochore formation (Fig. 5a). To further investigate this observation, we next measured the intrakinetochore levels of GFP-labelled reporter proteins Cse4, Mif2, Cnn1, Ame1, Mtw1 and Ndc80 in G1, S-phase, metaphase, and anaphase cells of *RIO1-AID* strains treated for 1 h with auxin or a mock. Compared to the mock-treated cells, the kinetochores in Rio1-depleted yeast contained enhanced levels of Cse4 and Mif2, and decreased numbers of Cnn1 and Ndc80. The levels of Ame1 and Mtw1 were not affected (Fig. 5b, Supplementary Fig. 6a–e). To examine whether the anomalous intrakinetochore levels of Cse4, Mif2, Cnn1, and Ndc80 resulted from an abnormal expression of these proteins, we first analysed our RNA-Seq data (Supplementary Data 2). They showed that the mRNA levels of these and all other known kinetochore proteins were not affected upon Rio1 depletion. Next, western blots revealed that the intracellular concentrations of Mif2, Cnn1, and Ndc80 did not change upon Rio1 depletion (Fig. 5c, Supplementary Fig. 7a). However, a 2.5-fold increase in Cse4 protein levels was measured in the cells depleted of Rio1 (Fig. 5c, Supplementary Fig. 7a), suggesting an augmented translation of its mRNAs and/or increased Cse4 protein stability. The elevated Cse4 protein levels may have caused its erroneous deposition into centromeric nucleosomes, and subsequently the faulty recruitment of kinetochore subunits, including Mif2, Cnn1, and Ndc80.

**Fig. 6:**
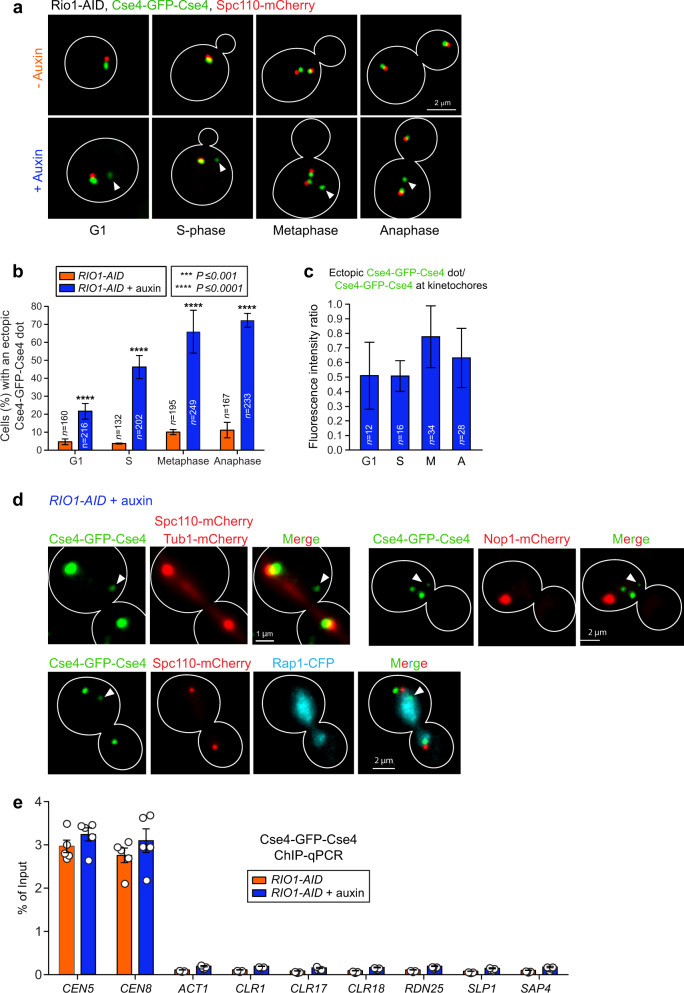
Rio1 prevents the formation of ectopic, immature kinetochores. **a** Representative images of Cse4-GFP-Cse4 localising ectopically (indicated by white arrowheads) in all cell cycle stages (identified by the position of mCherry-labelled spindle pole protein Spc110) of *RIO1-AID* cells treated with 500 µM auxin. **b** Percentage of *RIO1-AID* cells treated with 500 µM auxin or a mock that contained an unaligned, ectopic Cse4-GFP-Cse4 signal as identified in various cell cycle stages. The number of cells analysed (*n*) derived from three independent experiments. The data are shown as mean ± SD, and the *P*-values were calculated with the unpaired, two-tailed Student’s *t* test. **c** Ratio between the ectopic and spindle-aligned Cse4-GFP-Cse4 signals in various cell cycle stages. The number of cells analysed (*n*) derived from three independent experiments. The data are shown as mean ± SD. *P*-values were calculated with the unpaired, two-tailed Student’s *t* test. **d** Representative images of ectopic Cse4-GFP-Cse4 (indicated by white arrow heads) identified in *RIO1-AID* cells with marked spindle poles (Spc110-mCherry), genomic chromatin (Rap1-CFP), and nucleolus (Nop1-mCherry), treated for 1 h with 500 µM auxin. **e** Localisation of Cse4 at centromeres (CEN5, CEN8), at the *ACT1* locus, and at various loci and promoters to which Cse4 was shown to bind when overexpressed^27, 34^, as determined by ChIP-qPCR analysis. The singular data (white circles) were obtained from five independent biological experiments, and are shown combined as mean ± SD. *P*-values were calculated with the unpaired, two-tailed Student’s *t* test.

During our above imaging experiments, we observed in all cell cycle stages of Rio1-depleted cells a singular, ectopic signal for Cse4-GFP-Cse4 (Fig. 6a–d) as well as for Ame1-GFP (Supplementary Fig. 6e–g), which localised remotely from the kinetochores that clustered near the spindle poles (interphase, anaphase; Spc110-mCherry as the reference) or that were bi-oriented (metaphase). The other reporter proteins (Mif2, Cnn1, Mtw1, and Ndc80) did not localise ectopically (Supplementary Fig. 6a–d). The fluorescence intensities of the ectopic Cse4 and Ame1 signals were on average ~55% and ~15%, respectively, of those measured at the spindle pole-clustered or bi-oriented kinetochores (Fig. 6c, Supplementary Fig. 6g). The ectopic dot lied inside the nucleus but distant from the nucleolus (Fig. 6d). ChIP-qPCR analysis of Cse4-GFP-Cse4 at centromeres, at the *ACT1* locus (negative control), and at pseudo-centromeric sequences to which Cse4 binds when overexpressed^27,34^ did not identify Cse4 beyond centromeres in the Rio1-depleted cells (Fig. 6e), indicating that the ectopic signal represented (an) unbound, immature kinetochore(s) comprising Cse4 and Ame1, which Cse4 recruits^35^. Of note, yeast depleted of Rio1 in a synchronised or asynchronous culture did not arrest at the metaphase-anaphase transition possibly because the spindle assembly checkpoint requires structurally intact kinetochores.

**Fig. 7:**
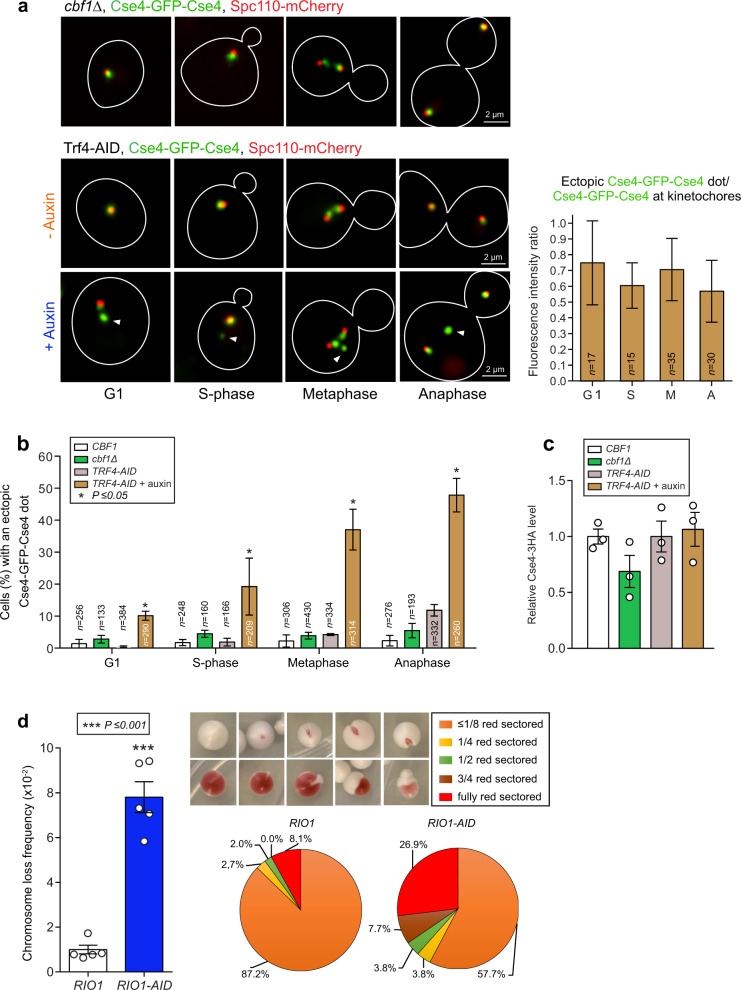
RNA turnover prevents anomalous kinetochore formation and chromosome loss. **a** Representative images of Cse4-GFP-Cse4 in a *cbf1Δ* mutant and in *TRF4-AID* cells treated with 500 μM auxin or a mock. Spindle pole marker mCherry-Spc110 was used to designate the cell cycle stages. Ectopic Cse4-GFP-Cse4 signals are indicated with a white arrowhead. Right plot: Ratio between the fluorescence signal of ectopic Cse4-GFP-Cse4, and that of Cse4-GFP-Cse4 localising to pole-clustered (G1, S-phase, anaphase) or bi-oriented (metaphase) kinetochores in *TRF4-AID* cells treated with 500 µM auxin. The number of cells analysed (*n*) derived from three replicate experiments. The data are shown as mean ± SD, *P*-values were calculated with the unpaired, two-tailed Student’s *t* test. **b** Percentage of wild-type (*CBF1*), *cbf1Δ*, and *TRF4-AID* yeast cells treated with a mock or 500 µM auxin that exhibited an ectopic Cse4-GFP-Cse4 signal in G1, S-phase (S), metaphase (M), and anaphase (A). The number of cells analysed (*n*) derived from three replicate experiments. The data are presented as mean ± SD. *P*-values were calculated with the unpaired, two-tailed Student’s *t* test. **c** Cse4-3HA levels measured by western blot in *CBF1*, *cbf1Δ*, and *TRF4-AID* yeast cells treated with 500 µM auxin or a mock. Pgk1 acted as the loading control and reference for relative quantifications (the blots are shown in Supplementary Fig. 7b). The measurements derived from three replicate experiments (*n* = 3) and were normalised to those measured in the wild-type cells (value = 1). Singular data (white circles) are combined as mean ± SEM. *P*-values were calculated with the unpaired, two-tailed Student’s *t* test. **d** *RIO1-AID* cells treated with 100 µM auxin for 6 h exhibited an 8-fold increase in chromosome loss, compared to wild-type yeast (*RIO1*). Colonies with red-colored sectors (representative images are shown), indicative of chromosome reporter fragment loss^36^, were counted, and normalised to those measured in the *RIO1* strain (value = 1). The data derived from five biological experiments (*n* = 5). The singular data (white circles) are combined as mean ± SEM. *P*-values were calculated with the unpaired, two-tailed Student’s *t* test. The distributions of generational chromosome loss frequencies are plotted in pie format.

To determine whether either derepressed transcription or reduced cen- and pericenRNA turnover may have contributed the most to kinetochore misassembly in yeast depleted of Rio1, we could not study separation-of-function *rio1* mutants; since these do not exist. Hence, we used a *cfb1Δ* strain (derepressed CEN transcription) and yeast depleted of TRAMP/nuclear exosome activity (*TRF4-AID* +auxin for 1 h; suffers from (peri)cenRNA build up) and probed the presence of ectopic Cse4-GFP-Cse4. While the latter was not observed in the *cbf1Δ* mutant, it was identified in all cell cycle stages in the Trf4-depleted cells (Fig. 7a, b). Of note, parallel western blot analyses revealed that Cse4 protein levels were not significantly altered in the cells lacking Cbf1 activity (*cbf1Δ*) nor in the Trf4-depleted cells (Fig. 7c, Supplementary Fig. 7b), suggesting that active (peri)cen transcript turnover is especially crucial for the correct inclusion of Cse4 in CEN nucleosomes and subsequent kinetochore formation.

Given the presence of misassembled and unbound kinetochores in the Rio1-depleted cells, we next measured the segregation fidelity of a centromere-harboring reporter chromosome fragment (colony-sectoring assay^36^) through three cell cycles (6 h) of *RIO1-AID* cells subjected to a lower level of Rio1 depletion, achieved by using 100 µM auxin (Supplementary Fig. 7c). We decided upon this approach to mitigate secondary effects caused by this multifunctional kinase (includes decreased ribosome biogenesis) during a 6 h treatment with 500 μM auxin. Importantly, lowering Rio1 depletion still generated increased cenRNA levels and the ectopic Cse4-GFP-Cse4 signal (Supplementary Fig. 7c). After growing the *RIO1* (control) and *RIO1-AID* strains in the presence of 100 µM auxin for 6 h, the cells were transferred onto agar-based medium lacking auxin, and then incubated for 5 days. We measured an eight-fold increase in chromosome missegregation (red-sectored colonies, Fig. 7d), corroborating that Rio1 activity prevents chromosome instability.

### RioK1 ensures correct cenRNA levels, faithful kinetochore assembly, and chromosome stability in human cells

**Fig. 8:**
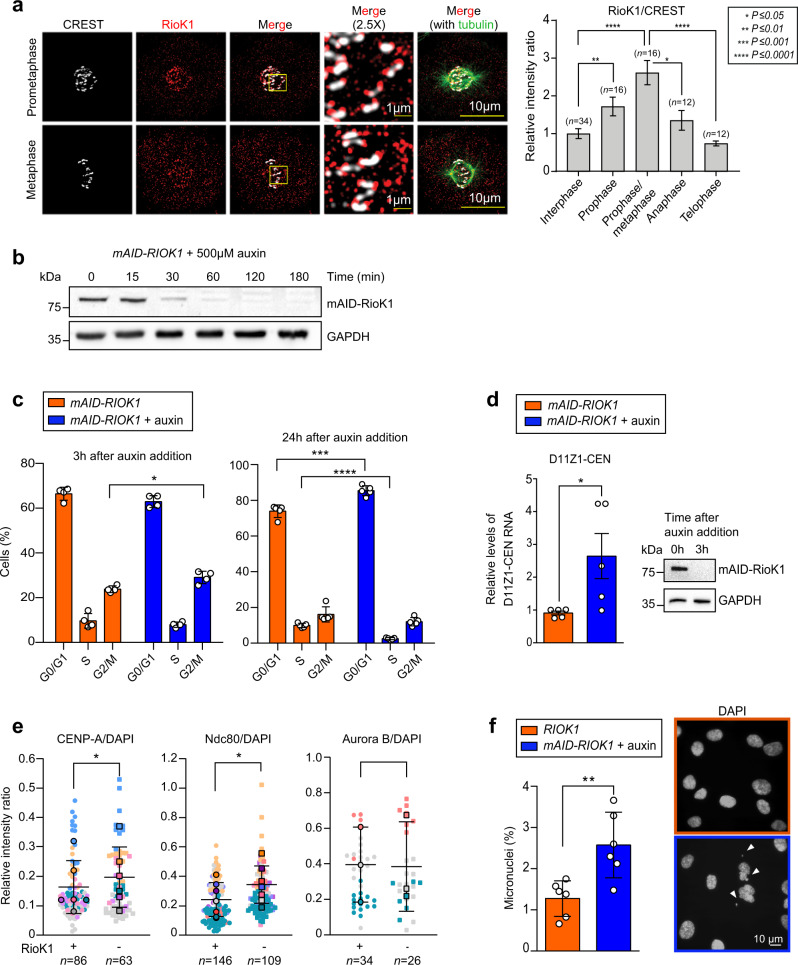
RioK1 ensures correct cenRNA levels, faithful kinetochore assembly, and chromosome stability in human cells. **a** Left: representative immunofluorescence (IF) images of RioK1 at kinetochores (CREST) in prometaphase and metaphase cells. Right: RioK1 levels at kinetochores (versus CREST) through the cell cycle. The numbers of cells analysed (*n*) derived from three replicate experiments. The data are shown as mean ± SD. *P*-values were calculated with the unpaired, two-tailed Student’s *t* test. Supplementary Fig. 8a shows IF images of RioK1 through the cell cycle. **b** Over-time levels of mAID-RioK1 and GAPDH (loading control) in *mAID-RIOK1* cells treated with 500 µM auxin. **c** Cell cycle analysis (FACS) of *mAID-RIOK1* cells treated with 500 µM auxin or a mock at mitotic entry. Cells were probed after 3 h or 24 h of treatment (four (*n* = 4) and five (*n* = 5) replicates per condition, respectively, Supplementary Fig. 8c). The data are shown as mean ± SD, *P*-values were calculated with the unpaired, two-tailed Student’s *t* test. **d**, Cen11 RNA levels (RT-qPCR) in *mAID-RIOK1* cells treated for 3 h with auxin or a mock (five replicative experiments, *n* = 5) were normalised to *GAPDH* mRNA levels, and then referenced to the cen11 RNA levels in the mock-treated cells. The data are shown as mean ± SEM. *P*-values were calculated with the unpaired, two-tailed Student’s *t* test. mAID-RioK1 and GAPDH levels before and after auxin treatment are shown. **e** CENP-A, Ndc80, and Aurora B levels at kinetochores formed de novo without RioK1 (Supplementary Fig. 8b). Protein levels were quantified by IF analysis and normalised to DNA (DAPI) levels. Protein:DAPI ratios are shown as SuperPlots. The number of cells analysed (*n*) derived from six (CENP-A), seven (Ndc80), or three (Aurora B) replicate experiments. *P*-values were calculated with the paired, two-tailed Student’s *t* test. **f** Cells (%) containing micronuclei formed during mitosis in *RIOK1* and *mAID-RIOK1* cells treated at mitotic entry with a mock or 500 µM auxin (3 h). The number of cells analysed (3387 and 3461; respectively) derived from six experiments (*n* = 6). The data are shown as mean ± SD. *P*-values were calculated with the unpaired, two-tailed Student’s *t* test. Right: representative images of DAPI-stained cells containing (orange) or lacking (blue) mAID-RioK1 activity. The white arrowheads indicate micronuclei.

To investigate whether orthologue RioK1 also regulates cenRNA levels and promotes correct CEN nucleosome and kinetochore formation in human cells, we first localised the kinase in hTERT-immortalised RPE-1 cells by immunofluorescence microscopy (anti-RioK1). RioK1 localised as speckles throughout the cytoplasm, indicative of its role in pre-40S ribosomal subunit maturation, but was identified also at centromeres/kinetochores (CREST antibodies as the reference). Its centromere presence peaked in prometaphase/metaphase, when human kinetochores assemble (Fig. 8a, Supplementary Fig. 8a). Next, using CRISPR/Cas9-based genome editing^37^, we homozygously labelled *RIOK1* with an N-terminal m(ini)AID tag^38^, allowing for its depletion within 3 h of treatment with 500 µM auxin (Fig. 8b). Next, *mAID-RIOK1* cells released synchronously after interphase enrichment (single thymidine treatment) followed by depletion of mAID-RioK1 upon entry in mitosis (Supplementary Fig. 8b) revealed an enrichment of the cells in G2/M after 3 h, and an accumulation in G0/G1 after 24 h of auxin treatment, as evidenced by DNA content analysis (Fig. 8c; Supplementary Fig. 8c). Asynchronous *mAID-RIOK1* cells treated with a mock or auxin for 3 h revealed a 2.5-fold increase in cenRNA levels (RT-qPCR) in the RioK1-depleted cells (Fig. 8d). Unfortunately, the redundant composition of the periCEN sequences did not allow for an unbiased quantification of pericenRNA levels. Next, the *RIOK1* and *mAID-RIOK1* cells, released from interphase, were treated with 500 µM auxin at mitotic entry. After 3 h, IF imaging revealed enhanced levels of CENP-A and Ndc80 in the kinetochores formed de novo in the absence of RioK1 activity. The intrakinetochore levels of Aurora B were not affected (Fig. 8e, Supplementary Fig. 8d). Western blots did not reveal any changes in the levels of these three reporter proteins (Supplementary Fig. 8e), indicating that depleting RioK1 disturbed CENP-A and Ndc80 recruitment to centromeres. While ectopic CENP-A signals were not observed following RioK1 depletion at mitotic entry, micronuclei were found to accumulate at the end of mitosis, indicating chromosome instability in cells lacking mitotic RioK1 activity (Fig. 8f).

## Discussion

Using Illumina’s NovaSeq 6000 100 bp paired-end sequencing platform (23–26 × 10^6^ mapped read pairs per sample), we set out to investigate the centromere transcripts present at physiological levels in *S. cerevisiae*. CenRNA reads were found in extremely low numbers (on average two per one thousand cells in an asynchronous culture), either as “short” reads with a median length of 231 nt, or as long reads with a median length of 4458 nt; both existing in a 1:1 ratio. The majority (78%) of the cenRNA reads were produced from within the upstream periCEN region, with the rest initiating inside the CEN sequence. While heterogeneous in composition and in length, most cenRNAs (48%) comprised the entire CEN core and extended beyond, whereas the other cenRNAs terminated inside the centromere’s CDEI (12%), CDEII (24%) or CDEIII (17%) elements. Most cenRNAs were generated from the upper (sense) strand. Structural work has revealed that two inner kinetochore CCAN networks assemble on the chromatin adjacent to the CEN core^39^. Possibly, the “arms” of the long and short cenRNAs could help to signal the CCAN proteins, and promote kinetochore assembly and stability.

Our observations that protein kinase/ATPase Rio1 downregulates cenRNA production by reducing local accessibility for RNAPII while also promoting cenRNA turnover, emphasises the commitment of cells to centromere silencing. The same is true, albeit at a different level, for the pericentromeres (within 500 bp from the CEN core sequences). We identified pericen transcripts as coding and noncoding. The noncoding pericenRNAs exceeded the cenRNA reads 390-fold, as they existed on average in 1 read pair per cell. In turn, the pericen mRNA reads exceeded the noncoding pericenRNA reads on average 10-fold. In contrast to the noncoding pericenRNA reads, the coding pericen mRNAs were not controlled by Rio1. Following Rio1 depletion, the noncoding pericenRNA read numbers increased 1.9-fold. However, in cells depleted of Rio1, the noncoding pericenRNA-to-cenRNA read ratio dropped with 20% (from 390:1 to 310:1) implying that Rio1 more strenuously restrains the cenRNA levels. While Rio1 downregulated the production and upregulated the degradation of the noncoding pericenRNAs, it was not involved in their maturation, as the median length of their transcript reads (225 nt) did not decrease upon Rio1 depletion (Fig. 1c).

The biological role(s) of the noncoding pericenRNAs remain(s) unknown in *S. cerevisiae*. In the fission yeast *Schizzosaccharomyces pombe*, the RNAi pathway -which did not evolve in *S. cerevisiae*- processes the pericen transcripts into 22 nt fragments, which recruit epigenetic enzymes that remodel, silence, and compact the periCEN domains^40,41^. This activity insulates the centromeres, promoting kinetochore recruitment onto them. In *S. cerevisiae*, the noncoding pericenRNAs could act similarly, despite their length (the shortest periCEN transcript reads identified in our *RIO1-AID* cultures treated with a mock or auxin, were 42 nt and 56 nt long, respectively). Our proteomic data revealed that Rio1 consistently interacted with the chromatin silencing histone deacetylases (HDAC) Sir2 and Sir3, Hos4 (subunit of the HDAC3 complex), and Sin3 (subunit of the Rpd3L and Rpd3S HDAC complexes). We previously showed that Rio1 promotes Sir2 recruitment to the rDNA to silence rRNA production in late anaphase^24^. Since Sir2 does not localise at centromeres^42^, we probed its presence by ChIP-qPCR analysis at pericentromeres (periCEN5) in *RIO1-AID* cells treated with a mock or auxin (Supplementary Fig. 7d). The enzyme was enriched at the pericentromere region adjacent to the CEN (within 200 bp). Importantly, Sir2 levels halved upon Rio1 depletion. In contrast, low Sir2 chromatin levels, which did not respond to Rio1 depletion, were measured at a noncoding periCEN5 region more distant from the CEN (at 1 kb) (Supplementary Fig. 7d). These findings suggest that Rio1 may support a Sir2-mediated silencing of the CEN-adjacent regions, an activity that may add to that of histone variant Htz1, which replaces H2.A in the CEN-flanking pericentromeric nucleosomes to silence them^27,43^.

Outside of S-phase, RNAPII resided -albeit in low numbers- also at centromeres and pericentromeres in metaphase (Fig. 3b), suggesting local, base-level transcription activity in mitosis. Human centromeres are transcribed in mitosis to maintain a functioning kinetochore and to promote centromeric cohesion^15,44,45^. In human cells, cohesin was shown to in turn regulate RNAPII transcription at centromeres^46^. In *S. cerevisiae*, the RSC chromatin remodeler complex supports cohesin in clustering centromeres^47^. Our purifications exposed strong interactions between Rio1 and chromatin remodelers RSC, Fun30, and SWI/SNF, the latter of which also support yeast CEN activity^47–51^. By targeting these proteins/complexes, Rio1 could control RNAPII access to and activity at the CEN and periCEN regions in early S-phase (cfr. the surge in local read start sites following Rio1 depletion) when cohesin becomes enriched at both domains. Noteworthy, Rio1 was originally identified in *Aspergillus nidulans* as a genetic suppressor of mutations in cohesin subunit Smc3 and cohesin regulator Pds5^52^, findings that we confirmed with *S. cerevisiae*^25^. Smc3 consistently co-purified with Rio1, further suggesting a functional relationship between Rio1 and cohesin activities. Within the context of transcription we wish to note that Rio1 consistently (three independent purifications) co-purified all five condensin subunits (Smc2, Smc4, Brn1, Ycs4, and Ycg1). Possibly, by managing local chromatin condensation, Rio1 could allow for low-level (peri)CEN transcription, also during mitosis.

Besides downregulating cen- and pericenRNA production, Rio1 also promotes their degradation via the 5’−3’ RNase Rat1, which targets these transcripts independently of 3’−5’ TRAMP/nuclear exosome. Of note, we could not specifically discriminate and study the degradation of long cenRNAs by Rat1 since their sequences partially overlap with the short cenRNAs and pericenRNAs, with the latter exceeding the long cenRNA read numbers 200-fold. Since Rat1 does not physically associate with (peri)centromeres^53^, it appears to promote cen- and pericenRNA turnover in an off-DNA manner. Rat1 acting upon cen- and pericenRNAs expands its known involvements in the management of RNA synthesis and activity. While Rio1 and Rat1 function in the same pathway, they were not found to physically interact in our current purifications and previous Y2H screens^25^. However, our proteomic studies identified proteins co-purifying with both Rio1 and Rat1, including known Rat1 regulators Grc3 and Las1^33^ that might act as intermediates.

Depleting Rio1 caused an untimely formation of kinetochores that were also structurally aberrant. This phenotype could result from dysregulated cen- and pericenRNA levels (as confirmed indirectly by cells depleted in TRAMP/nuclear exosome activity), and increased intracellular concentrations of Cse4 that become misdeposited in CEN nucleosomes. Rio1 consistently co-purified protein Pat1, which regulates Cse4 levels by preventing its ubiquitination and degradation^54,55^, as well as with chaperons Spt6^56^ and Rtt106^57^, which together with the SWI/SNF^50^ chromatin remodelling complex ensure correct Cse4/CENP-A deposition at centromeres only. Indeed, an involvement of Rio1 in regulating these activities may have contributed to the anomalous levels of Cse4 within CEN nucleosomes in yeast depleted of Rio1. Our current working model summarising Rio1 activity at yeast (peri)centromeres is show in Supplementary Fig. 9.

Besides regulating pre-rRNA synthesis and processing, and promoting pre-40S small ribosomal subunit maturation in yeast^24,30,58,59^ and humans^59,60^, Rio1/RioK1 also manages CEN activity and kinetochore formation in both species. Indeed, the levels of orthologue RioK1 peaked at centromeres in prophase/prometaphase, when human kinetochores assemble. Noteworthy, RioK1 relocated from the centromeres to the contractile ring in telophase, suggesting an involvement of this protein kinase/ATPase in cytokinesis, as recognized also in *S. cerevisiae*^25^. Depleting RioK1 likewise led to cenRNA build up and kinetochore misassembly. While intracellular CENP-A concentrations were not affected, CENP-A levels were elevated in CEN nucleosomes formed de novo in the absence of RioK1 activity, as observed also in *S. cerevisiae*. In yeast and human cells, intracellular Ndc80 levels were not affected following Rio1 and RioK1 depletion. However, the levels of Ndc80 at the outer region of yeast kinetochores were slightly decreased, whereas its levels in human kinetochores slightly increased. Given the differences in kinetochore composition (~100 and ~40 subunits in human and yeast kinetochores, respectively), kinetochore recruitment and organisation in both organisms; dysregulated CEN activity and CEN nucleosome formation following Rio1 and RioK1 depletion, may have differently affected kinetochore assembly in both species. Mapping a large number of kinetochore subunits (conserved and unique) in the absence of Rio1/RioK1 activity could validate this hypothesis.

Despite being an oncogenic protein kinase, Rio1/RioK1 biology remains poorly understood. Our previous work with yeast revealed that Rio1 responds to nutrient availability. Via its downstream gene and protein network, Rio1 up- or downregulates cell growth and division under rich or limited resources, respectively^25^. In *Drosophila*, RioK1 phosphorylates kinase mTor to activate the mTORC2 complex^61^, which integrates stress signals and nutrient availability to regulate metabolism, protein synthesis, growth, and proliferation. Our current findings that Rio1 and RioK1 promote CEN activity, kinetochore assembly and chromosome stability fits within the global context of this kinase/ATPase managing growth and cell division.

## Methods

### Yeast and human cell culture conditions

The *Saccharomyces cerevisiae* strains used in this study have the W303-1A genetic background, and are listed in Supplementary Table 1. All strains were created either by mating and tetrad dissection, followed by spore selection; by plasmid transformation, or by transformation and homologous recombination of PCR-generated deletion or epitope cassettes.

Cells were grown at 25 °C (25 × *g*) in YPDA (1% yeast extract, 2% peptone, 2% glucose, 0.3 mM adenine) or in complete synthetic medium (SCM; 0.67% yeast nitrogen base without amino acids (Difco, Cat # 291940), 2% glucose; 0.079% complete supplement mixture (MP Biomedicals, Cat # 4500-012), and 0.3 mM adenine).

To deplete Rio1, the *P*_*ADH1*_*-Oryza sativa (Os)TIR1-9Myc RIO1-AID* strain (referred to as *RIO1-AID* in the manuscript) was treated with 500 µM auxin (indole-3-acetic acid; Sigma, Cat # I3750) for 20 min, followed by treatment with 250 µM auxin for an additional 40 min.

hTERT-immortalised diploid non-tumor RPE-1 (retinal pigment epithelial) cells (ATCC, Cat # CRL-4000) expressing *OsTIR1-9Myc* was grown in DMEM/F12 medium w/o L-glutamine (ThermoFisher Scientific, Cat # 21331020) supplemented with 5% filter-sterilised fetal bovine serum (FBS; Sigma, Cat # F2442), 2.5% HEPES 1 M, 2% penicillin/streptomycin (10,000 U/ml; ThermoFisher Scientific, Cat # 15140122), 2% L-glutamine 4 mM, 0.12% sodium bicarbonate, 5 µg/ml plasmocin (Invivogen, Cat # ant-mpp), 100 µg/ml hygromycin B (Invivogen, Cat # ant-hg-1). The cells were cultured at 37 °C with 5% CO_2_, and were routinely passaged before reaching maximum confluence by removing the growth medium, followed by incubation (5 min, 37 °C) in 0.25% trypsin-EDTA (Sigma, Cat # T4049). Next, the detached and suspended cells were diluted five-fold with fresh growth medium, transferred into a new culture dish, and incubated for further growth.

### Construction of a homozygous *mAID-RIOK1* cell line and auxin-induced mAID-RioK1 depletion

To inducibly deplete RioK1 from hTERT-immortalised *OsTIR1-9Myc* expressing RPE-1 cells, we provided RioK1 with an N-terminal m(ini)AID degron^38^. Specifically, we cloned a promoter-free blasticidin resistance cassette in-frame with the sequence of self-cleaving viral peptide P2A, and the gene encoding the mAID protein from plasmid pMK347^62^ into a shuttle plasmid, generating donor plasmid p365. Using a forward 60nt oligomer comprising 40nt that are homologous to the *RIOK1* promoter sequence upstream of the *RIOK1* start codon, followed by the first 20nt of the blasticidin resistance cassette, and a reverse 60nt long oligomer comprising 40nt that are homologous to Exon 1 immediately downstream of *RIOK1*s’ start codon, followed by the last 20nt of the mAID sequence, we PCR-amplified the degron cassette from p365. Using an optimised protocol^37^, the cassette was then transfected into the cells together with recombinant *Streptococcus pyogenes* Cas9, and the crRNA-tracrRNA oligomer containing the crRNA spacer sequence that covers the start codon of *RIOK1*, in the presence of 1 mM NU7441, which inhibits DNA repair by non-homologous end-joining (Selleckchem, Cat # S2638). Homozygous insertions of the degron cassette at both *RIOK1* alleles were confirmed by PCR analysis, and the expression of mAID-RioK1 corroborated by western blot hybridisation (anti-RioK1, untagged cell line as the negative control). Western blot analysis revealed the depletion of mAID-RioK1 within 3 h of treatment with 500 µM auxin (Fig. 8b).

### RNA isolation

Yeast RNA was isolated from asynchronously growing cells or from cells first enriched in G1 (START) with α-factor pheromone (added at 3 µg/ml; 1.5 µg/ml; 1.5 µg/ml with 45 min intervals) and then synchronously released into the cell cycle following α-factor washout. Specifically, the cells were isolated from a 5 ml yeast culture sample (1000 × *g*; 3 min, 4 °C), washed with ice-cold AE buffer (50 mM sodium acetate pH 5.2, 10 mM EDTA pH 8.0), and stored at −80 °C. Next, RNA was extracted using phenol and chloroform^25^. Residual genomic DNA was removed (1 h, 37 °C) with DNase I (New England Biolabs, Cat # M0303L).

To isolate total RNA from hTERT-immortalised, and OsTIR1-expressing RPE-1 cells grown in 10 cm Petri dishes, TRIzol Reagent (ThermoFisher Scientific, Cat # 15596026) was used, according to the manufacturer’s instructions. All RNA was precipitated with 2.5 M LiCl, and residual DNA removed by incubation with DNase I (1 h, 37 °C; New England Biolabs, Cat # M0303L). All RNA was finally concentrated by precipitation (1 h, −80 °C) with 3 M NaAc plus 95% ethanol, and resuspended in 30 µl of nuclease-free water.

### RNA-Sequencing (RNA-Seq) and data analysis

All cytoplasmic and mitochondrial rRNAs were removed from one µg of purified yeast RNA (see above) with the QIASeq FastSelect rRNA Yeast (Qiagen, Cat # 334215). RNA-Seq libraries were synthesised with NEBNext Ultra II Directional Library Prep Kit for Illumina (NEB, Cat # E7760S) using Unique Dual Index UMI Adaptors RNA Set 1 (NEB # 7416). The cDNA libraries were quality controlled and then deep-sequenced in 100 bp paired-end mode on a NovaSeq 6000 Sequencing System (Illumina) at CIBIO’s Next Generation Sequencing Core facility. Reads from each sample were preprocessed by filtering for nucleotide quality (Q30 threshold), read length (minimum 36 nucleotides after trimming, no more than one N in a read), and removing sequencing adapters with Trimmomatic V.032^63^. Filtered reads were then aligned to the sacCer3 genome (assembly R64^64^) with STAR^65^, using the alignIntronMin 4 -alignIntronMax 5000 -outSAMmultNmax 1 parameters. Aligned reads were assigned to genes by using the–QuantMode GeneCounts option.

Centromere positions for each chromosome were obtained from the SGD database^66,67^. Reads mapping to the centromeres and pericentromeres (500nt upstream and downstream of the CEN core regions) were extracted to BED files for each sample using the bedtools intersect command^3,68^. We obtained reads deriving from the pericentromere but not overlapping the related centromere by performing a subtraction of reads overlapping the centromere from those overlapping within 500nt upstream/downstream of the centromere. To this end we used the bedtools intersect command with the -v option^3,68^. Start and end read positions were then extracted by a custom R (https://www.r-project.org/) script, computing their frequency and density at each nucleotide (as CPM-normalised counts of reads at the position) of the centromere and pericentromeric regions and thus obtaining the most frequently observed RNA fragments at each centromere and pericentromere. Protein-coding transcripts overlapping the so-defined pericentromeric regions were obtained by downloading the coordinates of sacCer3 genome transcripts from the SGD database^64^ and performing an intersection by means of the bedtools intersect command^3,68^. The potential read start site profile along the centromeric and pericentromeric regions was defined by counting reads only on their start positions to compute to the per-nucleotide density defined above. Annotated ORFs subject to altered Differential Gene Expression following Rio1 depletion are listed in Supplementary Data 2.

### RT-qPCR analysis

One µg of total yeast or total human RNA (DNaseI treated) was retrotranscribed into cDNA with the QuantiTect Reverse Transcription Kit (Qiagen, Cat # 205313), according to the manufacturer’s instructions. Next, 50 ng of cDNA was analysed by qPCR analysis using the iQ SYBER Green Supermix (Bio-Rad, Cat # 1708882) and specific primer pairs (300 nM) (Supplementary Table 2). All qPCR reactions were performed in triplicate on a CFX96 thermal cycler (Bio-Rad). The comparative cycle threshold (Cq) method was used for data analyses. The Log2 (2^−ΔΔCq^) data represent the relative changes in mRNA expression. *ACT1* (yeast) and *GAPDH* (human) acted as references to normalise gene expression.

### Cell cycle analyses

To enrich *S. cerevisiae* in G1, asynchronous yeast cultures (25 °C, 25 × *g*) were treated with α-factor (Genscript Biotech, Cat # RP01002) by adding it 3 times (3 µg/ml; 1.5 µg/ml; 1.5 µg/ml) with 45 min intervals. To synchronously release the cells into the cell cycle, the cultures were spun down, washed, and the cells resuspended in α-factor-free medium. To study the cell cycle of cells lacking Rio1, the G1-arrested *RIO1-AID* culture was treated with 500 µM auxin (Sigma-Aldrich, Cat # I5148) for 1 h before the α-factor release. The *RIO1-AID* strain was next released into the cell cycle using growth medium (YPDA or SCM) to which 250 µM auxin was added every 30 min. To study the consequence of Ndc10-1 inactivation on cenRNA levels through a synchronous cell cycle, the wild-type *NDC10* strain and the isogenic *ndc10-1* temperature-sensitive mutant were first grown exponentially in YPDA medium (25 °C, 25 × *g*), enriched in G1 with α-factor, and finally released in pre-warmed (37 °C) YPDA medium.

Yeast cell cycle stages were determined based on the budding state, spindle pole distribution (Spc110-mCherry) and/or spindle morphology (Tub1-mCherry or Tub1-GFP) during live-cell microscopy of cells imaged following fixation with 2% paraformaldehyde (25 °C, 2 min).

hTERT-immortalised, OsTIR1-expressing wild-type *RIOK1* and *mAID-RIOK1* RPE-1 cells were plated at 1.5 × 10^5^ cells in 9.5 cm^2^ dishes or at 2,5 × 10^5^ cells in 22,5 cm^2^ dishes containing complete DMEM/F12 and incubated at 37 °C with 5% CO_2_. After 24 h, at a 60–70% confluence, thymidine (Sigma, Cat # T1895), pre-heated at 65 °C, was added at a final concentration of 2 mM to enrich the cells in S-phase. After 24 h of thymidine treatment, the cells were washed three times with 1xPBS (phosphate-buffered saline) to remove thymidine and then placed back into the incubator with media containing 30 μM deoxycytidine (Sigma, Cat # D3897), allowing cells to progress synchronously up till mitotic entry. Next, the cell cultures enriched at mitotic onset were treated with 500 μM or a mock, and harvested by trypsinisation after 3 h or 24 h of growth for subsequent imaging, western blot, and fluorescence-activated cell sorting (FACS) analysis. The experimental scheme is graphically summarised in Supplementary Fig. 8b. For FACS analysis, hTERT-immortalised, *OsTIR1*-expressing RPE-1 cells (5 × 10^5^ cells) were harvested and centrifuged twice for 7 min at 500 × *g* at room temperature. The cell pellet was re-suspended in 300 μl of 1xPBS and then fixated in 700 μl pre-cooled ethanol 100%, added dropwise while gently vortexing. The samples were stored at −20 °C awaiting subsequent analysis. Next, the cells were thawed, washed twice with 1xPBS (1000 × *g*, 3 min, room temperature), each time aspirating and vortexing the pellet. To stain the chromosomes, the cell pellet was re-suspended in a 250 μl solution of 10 μg/ml propidium iodide (Invitrogen, Cat # P3566) and 100 μg/ml PureLink RNase A (Invitrogen, Cat # 12091021) diluted in 1xPBS. Samples were incubated for 30 min at 37 °C, and then stored at 4 °C. Samples were subsequently analysed on a Calibur or Aria III flow cytometer (BD Biosciences). Analysis of cell cycle progression was performed with ModFit LT 4.0 software (Verity Software House).

### Inhibiting RNA polymerase II activity

To study (peri)cenRNA levels in yeast with inactivated RNA polymerase II, the cells were enriched in G1 with α-factor (see above), and then synchronously released into the cell cycle in the presence of 3 µg/ml thiolutin (Cayman Chemicals, Cat # CAYM11350-5). The S-phase time point was collected after 20 min (at 30 min for the Rio1-depleted cells). To deplete Rio1 in parallel, the *RIO1-AID* strain was treated with both 500 µM auxin and 3 µg/ml thiolutin. Total RNA was isolated from the culture samples, retrotranscribed into cDNA, and analysed by RT-qPCR, as described earlier.

### ChIP-qPCR analysis

Chromatin immunoprecipitation (ChIP) experiments were performed as published^69^ with minor modifications. Specifically, yeast cultures (50 or 100 ml) were treated with 1% formaldehyde (Fisher Scientific, Cat # F79-500) for 20 min (25 °C, 25 × *g*). Next, the crosslinked cells were harvested, washed once with cold Tris-buffered saline (50 mM Tris-Cl pH 7.5, 150 mM NaCl), and once with cold double distilled water, before being drop-frozen in liquid N_2_, and stored at −80 °C. The next day, the cells were resuspended in 1.2 ml lysis buffer (50 mM HEPES pH 7.5, 150 mM NaCl, 1 mM EDTA pH 8.0, 1% Triton X-100, 0.1% sodium deoxycholate, 0.1% SDS) supplemented with 1% protease inhibitor cocktail (Sigma, Cat # P8215), 1 mM phenylmethylsulfonylfluoride (PMSF; ThermoFisher Scientific, Cat #36978), and 1% phosphatase inhibitor cocktail (Alpha Aestar, Cat # J61022). The resuspended cells were then ruptured with glass beads (Sigma, Cat # G8772) in a Fast Prep 24-5 G bead-beating and grinder-and-lysis instrument (MP Biomedicals) at 4 °C using the manufacturer’s standard settings for *S. cerevisiae* (4 repeats, 2 min intervals on ice). The obtained whole-cell extracts were next sonicated (Bioraptor Pico, Diagenode; 10 cycles of 15 s ON/30 s OFF) to shear the chromatin. 30 µl of the total chromatin was saved as input, the rest (300 µl) was incubated (2 h, 4 °C) with 4 µl of antibody (Ab; listed below) or without any antibody (noAb control), while 50 µl of SureBeads Protein G magnetic beads (Bio-Rad, Cat # 1614023) were incubated (2 h, 4 °C) with blocking solution (PBS, 0.02% Tween-20, 1% BSA, 4 µg salmon sperm DNA (Sigma, Cat # 438545-06-3). The magnetic beads were added to the chromatin samples, incubated (24 °C, 30 min), and washed twice with PBS plus 0.02% Tween-20. Next, the beads were transferred to a fresh Eppendorf tube, resuspended in 300 µl of elution buffer (1% SDS, 50 mM Tris-Cl pH 8.0, 10 mM EDTA), and incubated overnight (65 °C) to complete protein elution, and reverse the crosslinks. The input samples were similarly heat-treated. Following bead removal using a magnet holder, the supernatant was treated (30 min, 37 °C) with 2 µl of RNase A/T1 Mix (ThermoFisher Scientific, Cat # EN0551), and then with 20 µl Proteinase K (Euroclone, Cat # APA43920001) (2 h, 65 °C). Next, the chromatin was extracted with phenol, chloroform, and isoamyl alcohol (24:25:1 ratio; Sigma Cat # P3803). Following precipitation with 10 M NH_4_Ac and isopropanol, the DNA was resuspended in 140 µl nuclease-free water (Ambion, Cat # AM9937). Four µl of 1/50 diluted input, 4 µl of ChIP Ab samples, and noAb samples were analysed by qPCR analysis. The used primer pairs are listed in Supplementary Table 2.

The primary antibodies used in the ChIP experiments were anti-RNA polymerase II (mouse monoclonal, clone CTD4H8; Sigma, Cat # 05-623), living colors full-length anti-GFP (polyclonal rabbit, Takara Cat # 632592), and anti-Sir2 (goat polyclonal, Santa Cruz, Cat # sc-6666).

### Affinity purifications

To establish the Rio1 interactome, yeast lacking proteinase A activity (*pep4*∆) was transformed with a plasmid (*URA3* as the selection marker) expressing ProteinA-Rio1 from a *P*_*GAL1-10*_ promoter. For the negative control experiment, we transformed the same plasmid expressing untagged Rio1^59^. The strains were grown overnight (25 °C, 25 × *g*) in 500 ml of 0.5% glucose synthetic medium lacking uracil. In the morning, the cells were transferred to the same medium lacking any carbohydrate for 1 h, after which galactose (final 2%) was added. After 2 h, the cells were collected, rinsed with cold double distilled water, and then washed with and resuspended in 700 µl of cold breakage buffer (50 mM Tris-Cl pH7.5, 10% glycerol, 150 mM NaCl, 1 mM dithiothreitol, 0.4% NP-40, 1 mM PMSF, and 1% of protease inhibitor cocktail (Sigma, Cat # P8215). The cell suspension was then drop-frozen in liquid N_2_ and broken by mortar and pestle. The cell debris was removed (15,000 × *g*; 15 min, 4 °C), and the cleared lysate incubated (overnight, 4 °C) with 50 µl rabbit IgG agarose beads (Sigma, Cat # A2909) to capture ProteinA-Rio1 and associated proteins. The beads were then washed with PBS-T (PBS, 0.02% Triton X-100; Sigma Cat # T8787) and submitted to trypsin digestion and peptide analysis at CIBIO’s Mass Spectrometry and Proteomics facility to identify the co-purifying proteins. To establish the Rat1 interactome, Rat1 was endogenously tagged at its C-terminus with Protein A. The wild-type (untagged Rat1) yeast strain was used in parallel as the negative control. The cells were grown in YPDA medium (25 °C, 25 × *g*) till an OD_600_ of 1.2; washed, drop-frozen in liquid N_2_,and lysed using mortar and pestle, as described above, equally incubated with IgG-agarose beads, which were washed, and likewise subjected to mass spectrometry analysis. The PoteinA-Rio1, Rat1-ProteinA and negative control purifications were performed in triplicate, independent biological experiments (*n* = 3). (Supplementary Data 1).

### On-bead protein digestion and sample preparation for mass spectrometry analysis

The protein-bound IgG-agarose beads obtained from the ProteinA-Rio1 and Rat1-ProteinA purifications as well as those derived from the parallel negative-control purifications, were washed twice with 100 mM NH_4_HCO_3_ pH 8.3, and resuspended in 100 µl 6 M urea with 100 mM NH_4_HCO_3_. The bead-associated proteins were then reduced with 10 mM dithiothreitol (room temperature, 30 min), and alkylated with 20 mM C_2_H_4_INO in the dark (30 min). To digest the proteins, the beads were treated (room temperature, 4 h) with 0.75 μg Lys-C (Promega, Cat # VA1170) after which the solution was diluted four-fold in 50 mM NH_4_HCO_3_. An aliquot of 1.5 μg of sequencing-grade trypsin (Promega, Cat # V5111) was added to each sample before an overnight incubation at room temperature with gentle shaking. The beads were then separated from the supernatant by centrifugation, and the supernatant collected. Next, the digested peptides were acidified with 1% trifluoroacetic acid to a pH 2.5, desalted on C18 stage-tips, and resuspended in 20 μl of 0.1% formic acid buffer for LC-MS/MS analysis (see below).

### Mass spectrometry and liquid chromatography

The trypsin-generated peptides (see above) were separated chromatographically on an Easy-nLC 1200 system (ThermoFisher Scientific, USA) and loaded onto a reversed-phase column (Acclaim PepMap RSLC C18 column, 2 µm particle size, 100 Å pore size, I.D. 75 µm), heated at 40 °C with a two-component mobile phase system of 0.1% formic acid in water (buffer A), and 0.1% formic acid in acetonitrile (buffer B). Peptides were eluted using a gradient of 5% to 25% over 80 min, followed by 25% to 40% over 30 min, and 40% to 98% over 15 minutes, and kept at 98% over 15 min, a flow rate of 400 nl/min. Samples were injected in an Orbitrap Fusion Tribrid mass spectrometer (ThermoFisher Scientific), and data acquired in data-depended mode (2100 V). Full scans were performed at 120.000 FWHM resolving power (at 200 m/z) and an AGC target of 1x10e6. A mass range of 350–1100 m/z was surveyed for precursors, with first mass set at 140 m/z for fragments. Each full scan was followed by a set of MS/MS scans (HCD, collision energy of 30%) over 3 s cycle time at 150 ms maximum injection time (ion trap) and AGC target of 5 × 10e3. Peptides searches were performed in Proteome Discoverer 2.2 software (ThermoFisher Scientific) against the *S. cerevisiae* S288c FASTA file (UniProt, June 2021) and a database containing major common contaminants. Proteins were identified using a MASCOT search engine with a mass tolerance of 10 ppm for precursor and 0.6 Da for product. Trypsin was chosen as the enzyme with 5 missed cleavages, and static modification of carbamidomethyl (C) with variable modification of oxidation (M) and acetyl (protein N-term) were incorporated in the search. False discovery rate was filtered for <0.01 at PSM, peptide and protein level. The results were filters to exclude potential contaminants. Peak intensities were transformed into Log2 space. The data were normalised by the average of its abundance within each sample to account for variation in sampling volumes^18,70^. The mass spectrometry data are shown in Supplementary Data 1. Significant differences in peptide enrichment (*P* ≤ 0.05) were determined with the unpaired, two-tailed Student’s *t* test.

### Live-cell and immunofluorescence microscopy

For live-cell imaging experiments, yeast cells were grown in 2% glucose SCM supplemented with 0.3 mM adenine. The cells were mounted onto a 1.2% agarose cushion, and imaged on a Nikon Eclipse Ti2 microscope using a Plan Apochromatic 100× objective lens (NA 1.45), and an Andor Zyla 4.2 PLUS sCMOS camera. A Lumencor SpectraX illuminator with an excitation filter set acted as the light source, emission filters were from Chroma (ET525/50, ET605/70), and Semrock (FF01-515/588/700). Images were acquired across 20–30 Z-planes at 0.2 µm intervals, and analysed with ImageJ (FIJI) v2.3.0 (National Institutes of Health, USA). Z planes were projected with maximum intensity, background auto-fluorescence then subtracted, and kinetochore-GFP signals quantified using an 8-pixel ROI diameter.

Immunofluorescence imaging analysis of isolated and spread yeast nuclei was performed with 4 ml culture samples of the *6Myc-RIO1 NDC80-3GFP* strain. Specifically, each sample was centrifuged, and the yeast cell walls then digested (room temperature, 1 h) with 30 mg/ml zymolyase (Amsbio, Cat # 20 T). Next, the obtained spheroblasts were fixated onto a glass slide with 4% paraformaldehyde and 3.4% sucrose, and then spread with a glass rod. The slides were then hybridised with rabbit polyclonal anti-GFP antibody (ThermoFischer Scientific, Cat # A-6455), and mouse monoclonal anti-Myc antibody (9E10, Covance Cat # MMS-150R). CY3 AffiniPure donkey anti-mouse (Jackson ImmunoResearch, Cat # 715-165-151) or fluorescein isothiocyanate AffiniPure goat anti-rabbit (Jackson ImmunoResearch, Cat # 111-095-144) secondary antibodies were next added, while 1 µg/ml of DAPI (Sigma, Cat # D9542) was used to stain the DNA. The nuclei were captured with a BX51 wide-field fluorescence microscope (Olympus, USA). The images were deconvoluted (SoftWoRx), projected with maximum intensity, and the 6Myc-Rio1 and Ndc80-3GFP signals quantified using ImageJ v2.3.0.

For immunofluorescence microscopy analysis of human cells, hTERT-immortalised RPE-1 cells (RIKEN, Cat # CRL-4000, Japan) expressing *OsTIR1* were grown in medium and under conditions described above, on coverslips (Sigma, Cat # P4707). Next, the cells were washed with 1xPBS and fixed. To analyse RioK1 and CENP-A, the cells were fixed with 100% pre-cooled methanol for 10 min at −20 °C. To study Aurora B, the cells were fixed using a paraformaldehyde solution (3.7% paraformaldehyde, 30 mM sucrose, 1xPBS) for 10 min at room temperature, permeabilised with 0.1% Triton X-100/1xPBS (5 min, room temperature) and incubated with 0.1 M glycine (10 min, room temperature). To localise Ndc80, the cells were incubated with 0.3% Triton X-100/1xPHEM buffer (3 min; room temperature), washed in 1xPHEM and fixed in paraformaldehyde solution (15 min, room temperature). To study the localisation of CREST (centromere-binding proteins), and microtubule (α-tubulin), the protocols implemented were those used for the co-stained proteins (RioK1, CENP-A, Ndc80 or Aurora B), as described above.

All cells were next permeabilised using 0.3% Triton X-100/1xPBS (5 min, room temperature) and incubated with 0.1 M glycine (10 min, room temperature). After two washes with 1xPBS, the cells were treated (1 h, room temperature) with PTB blocking solution (1xPBS, 0.05% Tween, 3% bovine serum albumin (BSA)), and then stained with a primary antibody diluted in PTB (overnight, 4 °C) in a humidification box. The following primary antibodies were used: anti-Aurora B (rabbit polyclonal, 1:1000, Abcam, Cat # ab2254), anti-CENP-A (mouse monoclonal, 1:100; Abcam, Cat # ab13939), anti-centromere (derived from CREST patient serum; human, 1:50; Antibodies Incorporated, Cat # 15-234), anti-Ndc80 (rabbit polyclonal,1:200; Life technologies, Cat # PA5-78319), anti-RioK1 (mouse polyclonal, 1:500; Abnova Cat # H00083732-B02P), anti-alpha tubulin (mouse monoclonal, 1:1000, clone B512; Sigma, Cat # T5168). After three washes in 1xPBS-0.05% Tween 20, the cells were incubated with a secondary antibody conjugated to a fluorophore (30 min, room temperature). The following secondary antibodies were used: goat anti-mouse Alexa Fluor 647 (1:500; Invitrogen, Cat # A-21236), goat anti-mouse Alexa Fluor 555 (1:500; Invitrogen, Cat # A-21425), goat anti-rabbit Alexa Fluor 647 (1:500; Invitrogen, Cat # A-21245), goat anti-human Alexa Fluor 488 (1:500; Invitrogen, Cat # A-11013). Next, the cells were washed with 1xPBS-0.05% Tween 20. Finally, DNA was stained with 0.5 μg/ml DAPI (Sigma, Cat # D9542) for 3 min, and washed twice with 1xPBS. Slides were mounted with Prolonged Gold anti-fade reagent (Invitrogen, Cat # P36934).

The cells were imaged with a confocal spinning disk Nikon Eclipse Ti microscope using an immersion oil 60× or 100× objective with NA 1.45 (Nikon Corporation, Germany). All images were acquired as Z-stacks and processed with ImageJ v2.3.0. Aurora B, CENP-A, and Ndc80 signals were quantified (IntDen; average signal intensity over the area) within ROIs comprising the DAPI-stained DNA. For each cell, protein signal intensities were analysed within the DNA volume using ImageJ’s 3D suite plugin. Protein signals were then normalised to DAPI intensity. Z-stack images were deconvoluted with Huygens Profession software (v3.0.3, Scientific Volume Imaging) to reverse optical distortions and improve spatial resolution.

### Western blot hybridisation

To probe yeast proteins by western blot hybridisation, yeast cells were collected, treated with 5% trichloroacetic acid, resuspended in lysis buffer (50 mM Tris-Cl pH 7.5, 1 mM EDTA.Na2, 50 mM dithiothreitol, 60 mM β-glycerophosphate, 0.1 mM Na_3_VO_4_, 5 mM NaF) and provided with 1% protease inhibitor cocktail (Sigma, Cat # P8215). The resuspended cells were broken with the Fast Prep 24-5 G instrument (MP Biomedicals) applying the standard settings indicated for *S. cerevisiae* (4 repeats with 2 min intervals on ice). The lysates were cleared by centrifugation (14,000 × *g*; 10 min, 4 °C), and 4x SDS-PAGE sample loading buffer (200 mM Tris-Cl pH 6.8, 400 mM dithiotreitol, 8% SDS, 0.4% bromophenol blue, 40% glycerol) was added. The samples were then boiled (5 min), and the proteins separated by SDS-polyacrylamide gel electrophoresis. Next, the proteins were transferred onto Immobilon-P PVDF membranes (Millipore, Cat # IPVH00010), and the membranes incubated with primary anti-AID (mouse monoclonal, 1:1000; Covance, Cat # CAC-APC004AM-T), anti-Pgk1 (mouse monoclonal, 1:5,000; Life Technologies, Cat # 459250), anti-Myc (mouse monoclonal, 1:1,000; Covance, Cat # MMS-150R), anti-HA (mouse monoclonal, 1:1,000; Covance, Cat # MMS-101P) and peroxidase anti-peroxidase (rabbit, 1:1,000; Merck, Cat # P1291-500UL) antibodies. Secondary polyclonal horseradish peroxidase-conjugated goat anti-mouse (1:2,000; Dako, Cat # P0161) antibody was used to visualise the proteins with ECL SuperSignal West Femto solution (ThermoFisher Scientific, Cat # 34094). Images were acquired with a Chemidoc XRS+ system (Bio-Rad). Images were analysed with Image Lab software (Bio-Rad), and the signals quantified with Image J v2.3.0.

To probe human proteins by western blot hybridisation, whole-cell hTERT-immortalised, *OsTIR1*-expressing RPE-1 cell lysates were prepared with lysis buffer (10 mM Tris-Cl pH7.4, 150 mM NaCl, 0.5% NP-40, 0.5% sodium deoxycholate) containing a 1% protease inhibitor cocktail (Sigma, Cat # P8340). Thirty μg of total protein, as measured by the bicinchoninic acid assay (ThermoFisher Scientific, Cat # 23227) were submitted to SDS-polyacrylamide gel electrophoresis. The separated proteins were then transferred onto a 0.2 µm nitrocellulose membrane using the Trans-Blot Turbo Transfer System (Bio-Rad). As primary antibodies we used: anti-Aurora B (rabbit polyclonal, 1:500; Abcam, Cat # ab2254), anti-CENP-A (mouse monoclonal, 1:500,ThermoFisher Scientific, Cat # MA1-20832), anti-Ndc80 (rabbit polyclonal, 1:500; Life technologies, Cat # PA5-78319), anti-RioK1 (mouse polyclonal, 1:500; Abnova, Cat # H00083732-B02P). As the control for protein loading, we probed glyceraldehyde-3-phosphate dehydrogenase (GAPDH) (rabbit monoclonal, 1:5,000; clone 14C10, Cell Signaling, Cat # 2118). As secondary antibodies we used polyclonal horseradish peroxidase-conjugated anti-mouse IgG (1:5,000; Dako, Cat # P0161), and polyclonal horseradish peroxidase-conjugated anti-rabbit IgG (1:5,000; Dako, Cat # P0448). Protein signals on the membrane were detected with ECL SuperSignal West Femto solution (ThermoFisher Scientific, Cat # 34094) on a Chemidoc XRS+ system (Bio-Rad). Images were analysed with Image Lab software (Bio-Rad), and the signals quantified with Image J v2.3.0. Supplementary Fig. 10 shows all western blots and the specific sections (indicated by red rectangles) that are shown in the main figures.

### Yeast chromosome loss assay

The colony sectoring assay^36^ was used to measure the transmission of a CEN3-harbouring chromosome fragment carrying *SUP11-1*, which encodes an ochre (nonsense stop codon)-suppressing *tRNA*. The parental wild-type (*RIO1*) and *RIO1-AID* strains are auxotrophic for adenine due to an ochre mutation in *ade2* (*ade2-1*), which is relieved by *SUP11* tRNA activity. Loss of the chromosome fragment hence prevents the latter, causing red intermediate P-ribosyl-aminoimidazole to accumulate (is not converted by Ade2-1), as visually evidenced by red-colored cells (sectors) within a colony. Specifically, the *RIO1* and *RIO1-AID* strains carrying the chromosome fragment were grown overnight (25 °C, 25 × *g*) in 2% glucose, 0.2% CSM lacking adenine (Formedium, Cat # DSCK022). Next, the cells were diluted in 2% glucose, 0.2% CSM containing adenine, and treated with 100 µM auxin for two cell divisions (6 h, 25 °C, 25 × *g*) after which the cells were plated (at 100–150 cells per Petri dish) on 2% glucose, 0.2% CSM lacking adenine and supplemented with 2.2% agar, 0.1 mM adenine, and 0.8 mM tryptophan. After 5d of growth (room temperature), all colonies (white, red, and red-sectored; examples are shown in Fig. 7d) were counted and the chromosome loss frequency calculated.

### Statistical data analysis

Data averages, standard deviations and the statistical relevance of independence between discrete datasets (*P*-value, two-tailed Student’s *t* tests) were calculated using GraphPad Prism 6.01.

### Reporting summary

Further information on research design is available in the Nature Portfolio Reporting Summary linked to this article.

## Supplementary information

Supplementary Information file

Description of Additional Supplementary Files

Supplementary Data 1

Supplementary Data 2

Reporting Summary

## Data Availability

The data that support this study are available from the corresponding author upon reasonable request. Filtered RNA reads were aligned to the sacCer3 genome (assembly R64). The RNA-Seq data are available via the Gene Expression Omnibus (GEO, NCBI) with accession number GSE218602. The proteomic data are available via ProteomeXchange with identifier number PXD038337. Source data are provided with this paper.

## Source data

Source Data
