# Supplementary material for: Rio1 downregulates centromeric RNA levels to promote the timely assembly of structurally fit kinetochores
Source: Nat Commun. 2023 Jun 1;14:3172. doi: 10.1038/s41467-023-38920-9 (PMC10235086; doi:10.1038/s41467-023-38920-9)
Supplement: Supplementary file 1 — Supplementary Information file [file 41467_2023_38920_MOESM1_ESM.pdf]

Supplementary Figure 1

a cenRNA + coding and noncoding pericenRNA reads

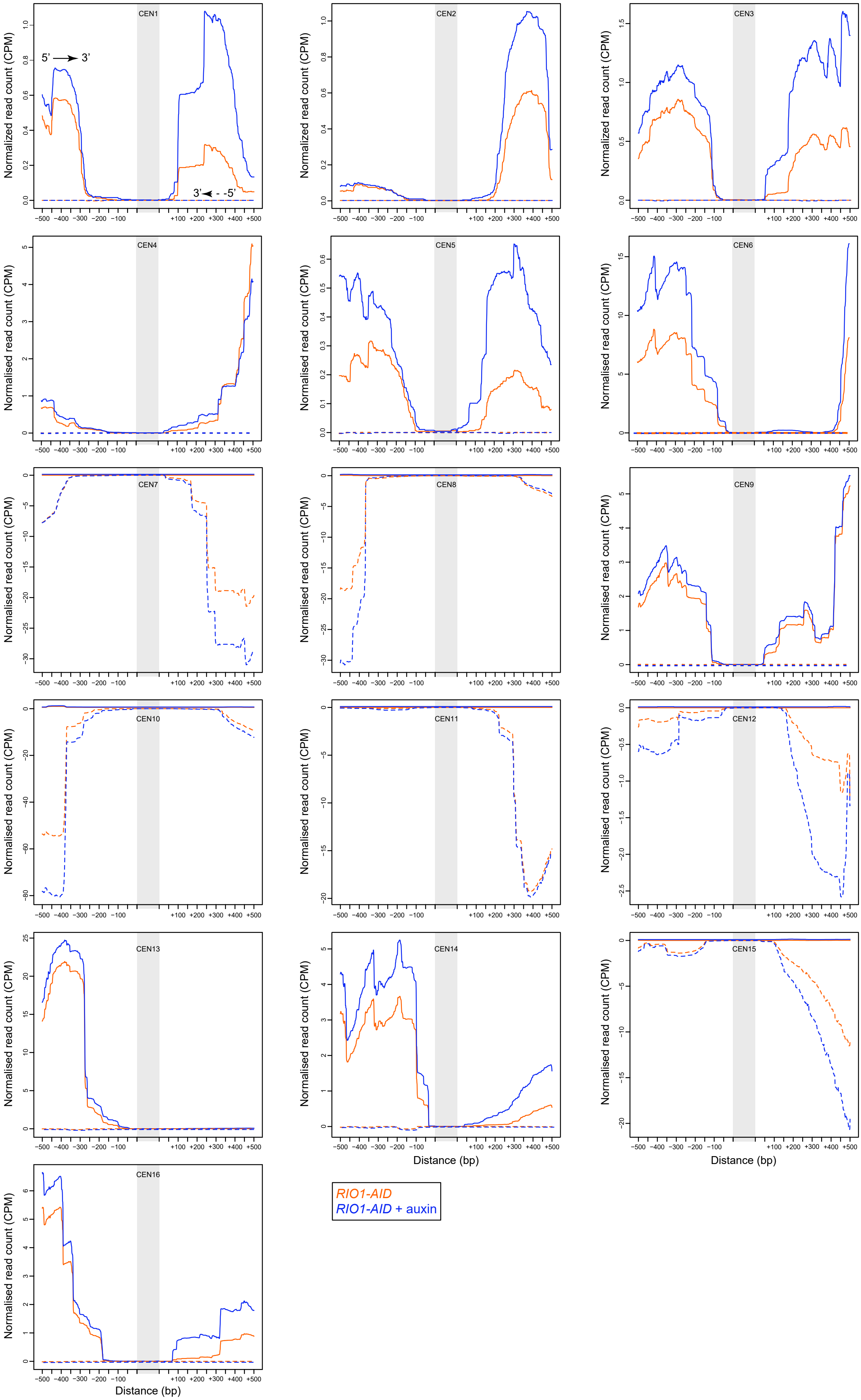

Supplementary Figure 1 (continued)

b cenRNA + noncoding pericenRNA reads

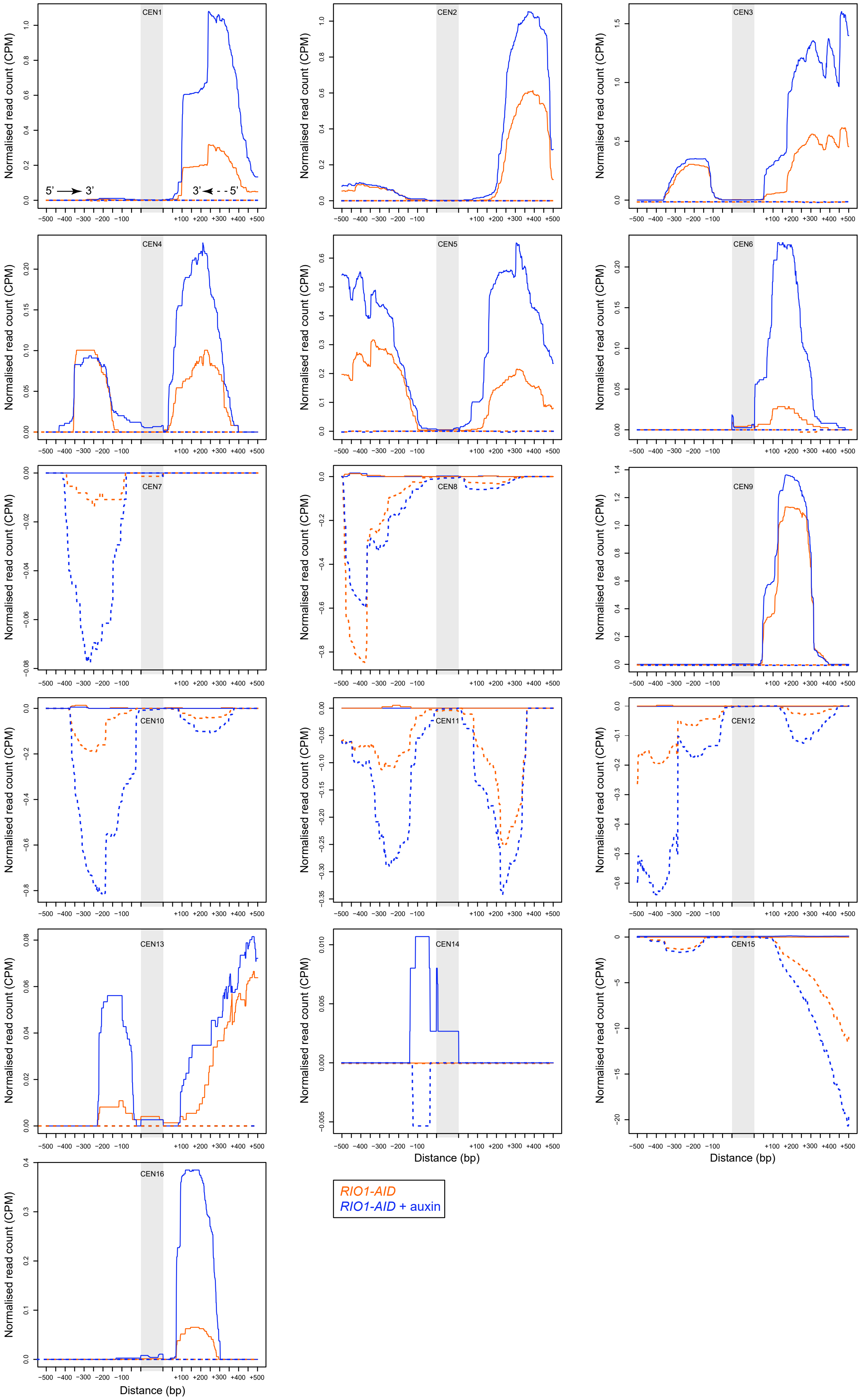

Supplementary Fig. 1 │ The transcription landscape at yeast centromeres and pericentromeres. **a**, Number of cen-, coding and noncoding pericenRNA read pair counts deriving from each chromosome and tallied from three independent biological experiments (*n*=3). The CEN core sequence is indicated by the grey column. The full lines indicate the reads deriving from the upper (sense, plus) strand, the dashed lines those deriving from the lower (anti-sense, minus) strand. Orange lines: *RIO1-AID* cells treated with a mock for 1h, blue lines: *RIO1-AID* cells treated with 500μM auxin for 1h. The plots comprise the 500bp periCEN regions surrounding the CEN core sequence. **b**, Number of cen- and noncoding pericenRNA reads deriving from each chromosome and tallied from three independent biological experiments (*n*=3). For description of the plots, see panel **a**.

Supplementary Figure 2

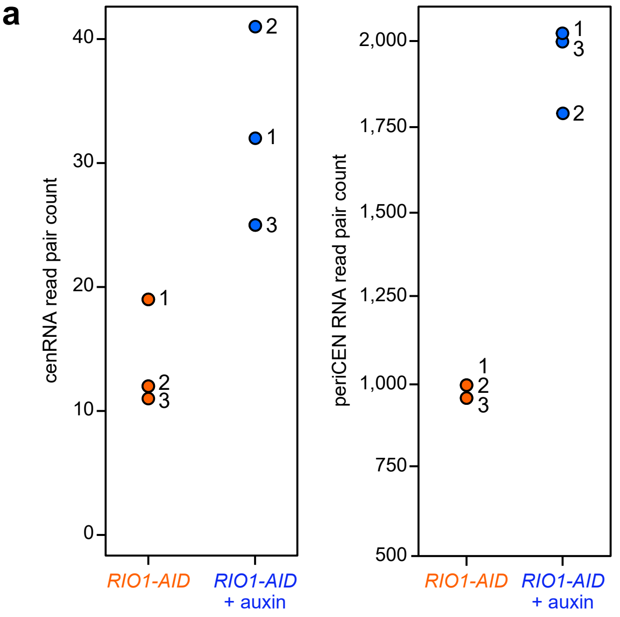

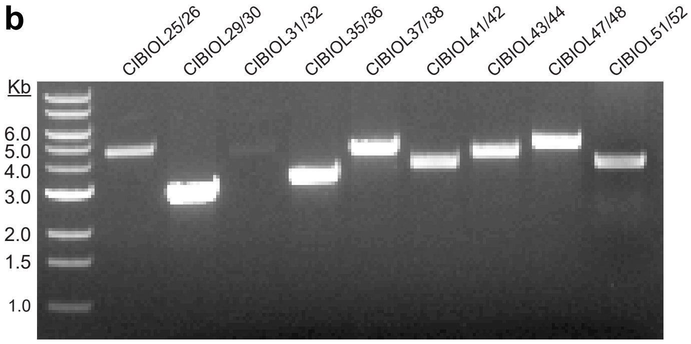

Chromosome	Read pair start (nt)	Read pair end (nt)	Read pair length (nt)	Oligomer pair
XIII	267,408	272,270	4,862	CIBIOL25/26
VII	494,693	497,653	2,960	CIBIOL29/30
II	237,003	241,533	4,530	CIBIOL31/32
II	236,905	240,252	3,347	CIBIOL35/36
XI	436,798	441,253	4,455	CIBIOL37/38
IV	447,821	451,568	3,747	CIBIOL41/42
XIV	626,377	630,672	4,295	CIBIOL43/44
XI	436,323	441,080	4,757	CIBIOL47/48
VIII	104,969	108,810	3,841	CIBIOL51/52

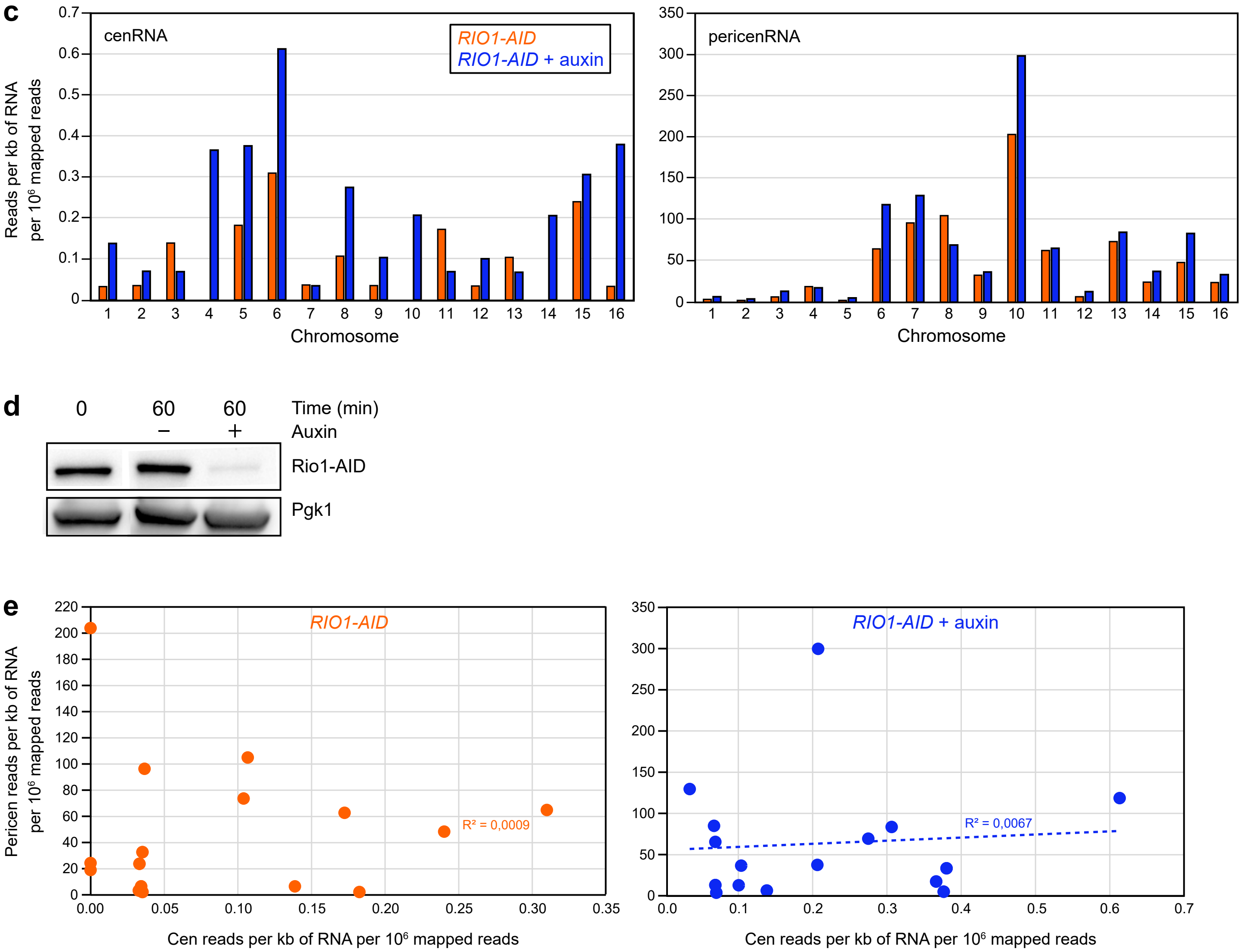

Supplementary Figure 2 (continued)

Supplementary Fig. 2 │ Characterisation of yeast cen and pericen transcripts. **a**, The number of cen- and pericenRNA reads identified in three independent experiments (*n*=3) represented by the three circles. Orange: *RIO1-AID* cells treated with a mock for 1h, blue: *RIO1-AID* cells treated with 500µM auxin for 1h. **b**, Agarose gel revealing specific cen transcripts identified by our RNA-Seq analyses and confirmed by PCR analysis of cDNA deriving from asynchronous *ndc10-1* cells, shifted for 3h from 25°C to 37°C. The flanking table indicates the chromosome number, the bioinformatically revealed cenRNA read start and termination sites, the predicted length of each read pair, and the names of the primer pairs used in the PCR reactions. The primer sequences are listed in Supplementary Table 2. **c**, The number of cen-, coding and noncoding pericenRNA read pairs deriving from each chromosome as measured by RNA-Seq analysis of *RIO1-AID* cells treated for 1h with a mock (orange bars) or 500µM auxin (blue bars). The data represent tallies from three independent biological experiments (*n*=3). **d**, Western blot of Rio1-AID (anti-AID) in the *RIO1-AID* strain treated for 1h with a mock or 500µM auxin. Protein Pgk1 (3-phosphoglycerate kinase 1, anti-Pgk1) served as the loading control. **e**, Plots revealing the absence of correlation between cen- and pericenRNA read pair numbers deriving from each of the sixteen chromosomes in *RIO1-AID* cells treated for 1h with a mock (upper plot) or 500µM auxin (lower plot).

Supplementary Figure 3

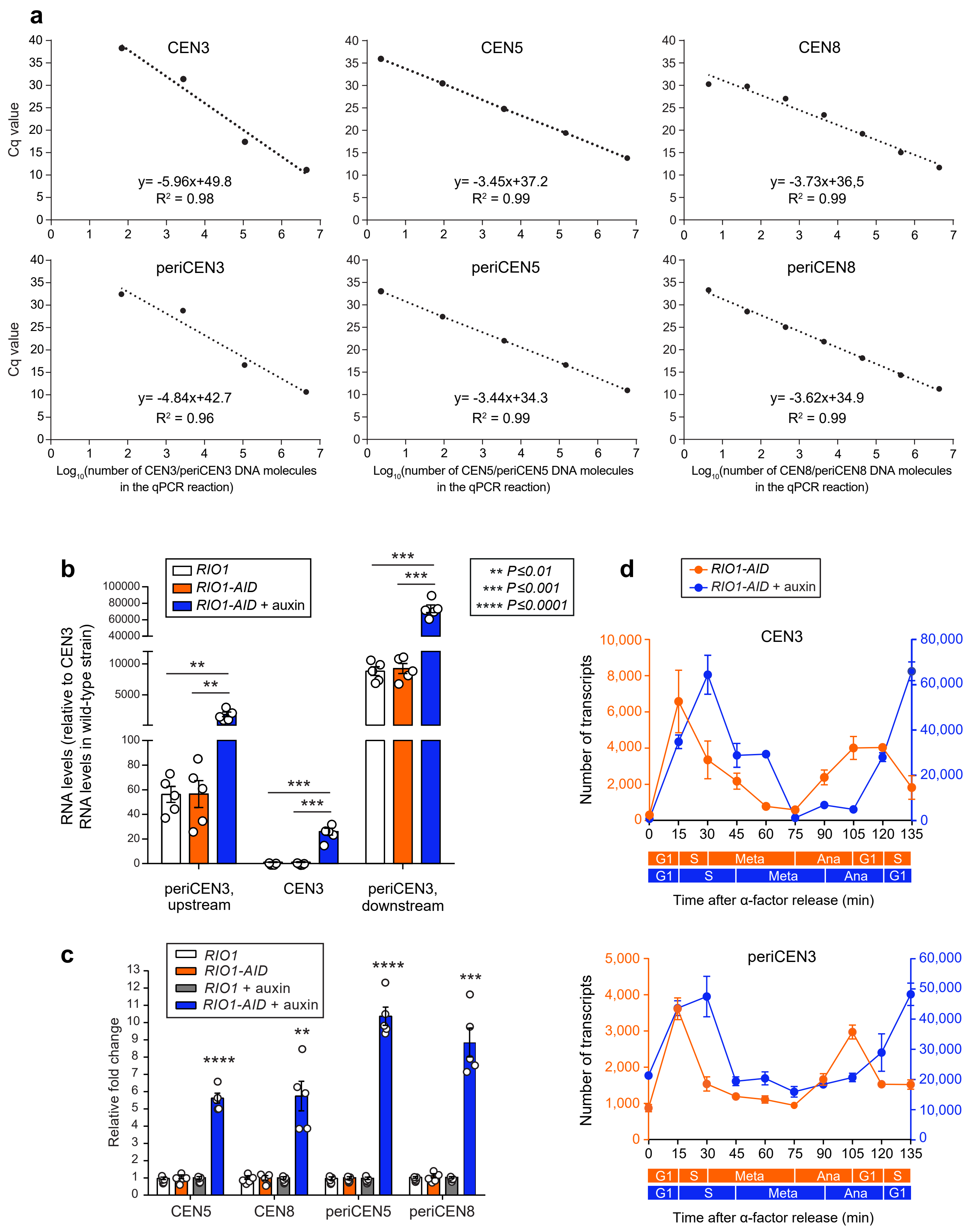

Supplementary Figure 3 (continued)

e

	CEN5	periCEN5	CEN8	periCEN8	CEN3	periCEN3
T0 *RIO1-AID* vs T0 *RIO1-AID* + auxin	ns	****	ns	****	***	***
T15 *RIO1-AID* vs T30 *RIO1-AID* + auxin	**	**	**	****	**	ns
T30 *RIO1-AID* vs T45 *RIO1-AID* + auxin	*	**	****	****	*	ns
T45 *RIO1-AID* vs T60 *RIO1-AID* + auxin	*	**	*	****	****	*
T60 *RIO1-AID* vs T75 RIO1-AID + auxin	*	**	ns	****	ns	ns
T75 *RIO1-AID* vs T90 *RIO1-AID* + auxin	ns	**	**	****	***	***
T90 *RIO1-AID* vs T105 *RIO1-AID* + auxin	**	***	*	***	**	ns
T105 *RIO1-AID* vs T120 *RIO1-AID* + auxin	**	**	**	****	***	ns
T120 *RIO1-AID* vs T135 *RIO1-AID* + auxin	*	***	*	****	***	**

f

	CEN5	CEN8
T0 *NDC10* vs *ndc10-1*	****	**
T15 *NDC10* vs *ndc10-1*	**	**
T30 *NDC10* vs *ndc10-1*	***	**
T45 *NDC10* vs *ndc10-1*	**	*
T60 *NDC10* vs *ndc10-1*	**	***
T75 *NDC10* vs *ndc10-1*	**	***
T90 *NDC10* vs *ndc10-1*	**	*

*	*P*≤0.05
**	*P*≤0.01
***	*P*≤0.001
****	*P*≤0.0001
ns	not significant

Supplementary Fig. 3 │ Quantification of cen- and noncoding pericenRNAs. a, Standard curves used to calculate cen- and noncoding pericenRNA numbers. CEN and periCEN DNA sequences were amplified from a wild-type yeast genome by qPCR (for primer pairs see Supplementary Table 2). periCEN3, upstream; periCEN5, downstream, periCEN8, upstream. The PCR-amplified DNA products were purified, quantitated (ng/µl), and their concentrations converted to molarity. Serial dilutions of the purified DNA were amplified by qPCR, and Cq reaction values plotted against the input DNA concentrations, yielding a 1st-order regression curve. Next, cen and pericen cDNAs were generated from various strains grown under different conditions (three replicates, *n*=3). The Cq values of the qPCR reactions were intrapolated to obtain the number of transcript molecules from the above regression curves. **b**, Levels of cen3 and pericen3 transcripts measured in *RIO1-AID* cells treated for 1h with a mock or 500µM auxin, referenced to *ACT1* mRNA levels, and normalised to the cen3 transcript levels measured in the wild-type (*RIO1*) (value = 1). The singular data (white circles) derived from five experiments (*n*=5) and are shown as means ± SEM. **c**, Levels of cen5, cen8, pericen5, and pericen8 transcripts in the *RIO1* or *RIO1-AID* strains treated for 1h with a mock or 500µM auxin. The transcripts were measured relative to those quantitated in the *RIO1* strain treated with a mock (value = 1). The singular data (white circles) derived from five experiments (*n*=5) and are shown as means ± SEM. **d**, Cen3 and pericen3 RNA numbers through the cell cycle of the *RIO1-AID* strain treated with a mock or 500µM auxin. Transcript levels were normalised to those of *ACT1* mRNA, and the number of transcript molecules derived from the curves in panel **a**. G1 = G1 phase, S = S-phase, M = metaphase, A = anaphase. All data were obtained from three replicates (*n*=3). Error bars represent SD. **e**, Confidence levels (*P*-values) for the data in Fig. 2b, and Supplementary Fig. 3d as calculated with the unpaired, two-tailed student t-test. **f**. Confidence levels (*P*-values) for the data in Fig. 2d, as calculated with the unpaired, two-tailed student t-test.

Supplementary Figure 4

a

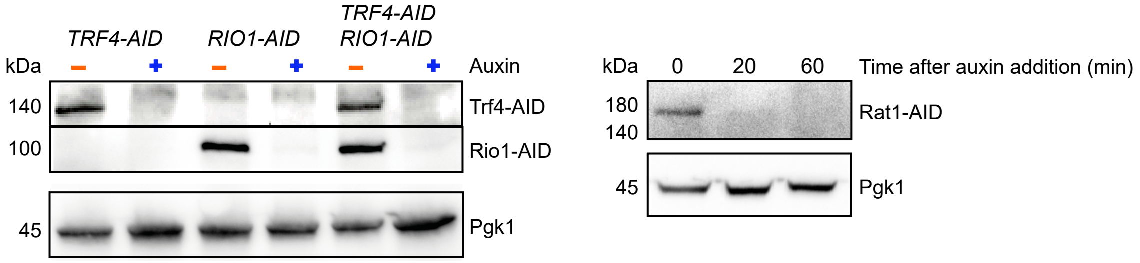

b

CEN5	*RIO1-AID, TRF4-AID, RAT1-AID*	*RIO1-AID, TRF4-AID*	*RIO1-AID, RAT1-AID*
RIO1-AID	****	****	n.s.
RAT1-AID	****	—	*
TRF4-AID	****	****	—
RIO1-AID, RAT1-AID	****		
RIO1-AID, TRF4-AID	n.s.		

CEN8	*RIO1-AID, TRF4-AID, RAT1-AID*	*RIO1-AID, TRF4-AID*	*RIO1-AID, RAT1-AID*
RIO1-AID	****	*	n.s.
RAT1-AID	****	—	n.s.
TRF4-AID	****	****	—
RIO1-AID, RAT1-AID	****		
RIO1-AID, TRF4-AID	****		

*	*P* ≤0.05
**	*P* ≤0.01
***	*P* ≤0.001
****	*P* ≤0.0001
n.s.	not significant
—	not applicable

periCEN5	*RIO1-AID, TRF4-AID, RAT1-AID*	*RIO1-AID, TRF4-AID*	*RIO1-AID, RAT1-AID*
RIO1-AID	****	****	****
RAT1-AID	n.s.	—	*
TRF4-AID	n.s.	****	—
RIO1-AID, RAT1-AID	*		
RIO1-AID, TRF4-AID	****		

periCEN8	*RIO1-AID, TRF4-AID, RAT1-AID*	*RIO1-AID, TRF4-AID*	*RIO1-AID, RAT1-AID*
RIO1-AID	n.s.	n.s.	n.s.
RAT1-AID	**	—	***
TRF4-AID	**	****	—
RIO1-AID, RAT1-AID	n.s.		
RIO1-AID, TRF4-AID	n.s.		

c

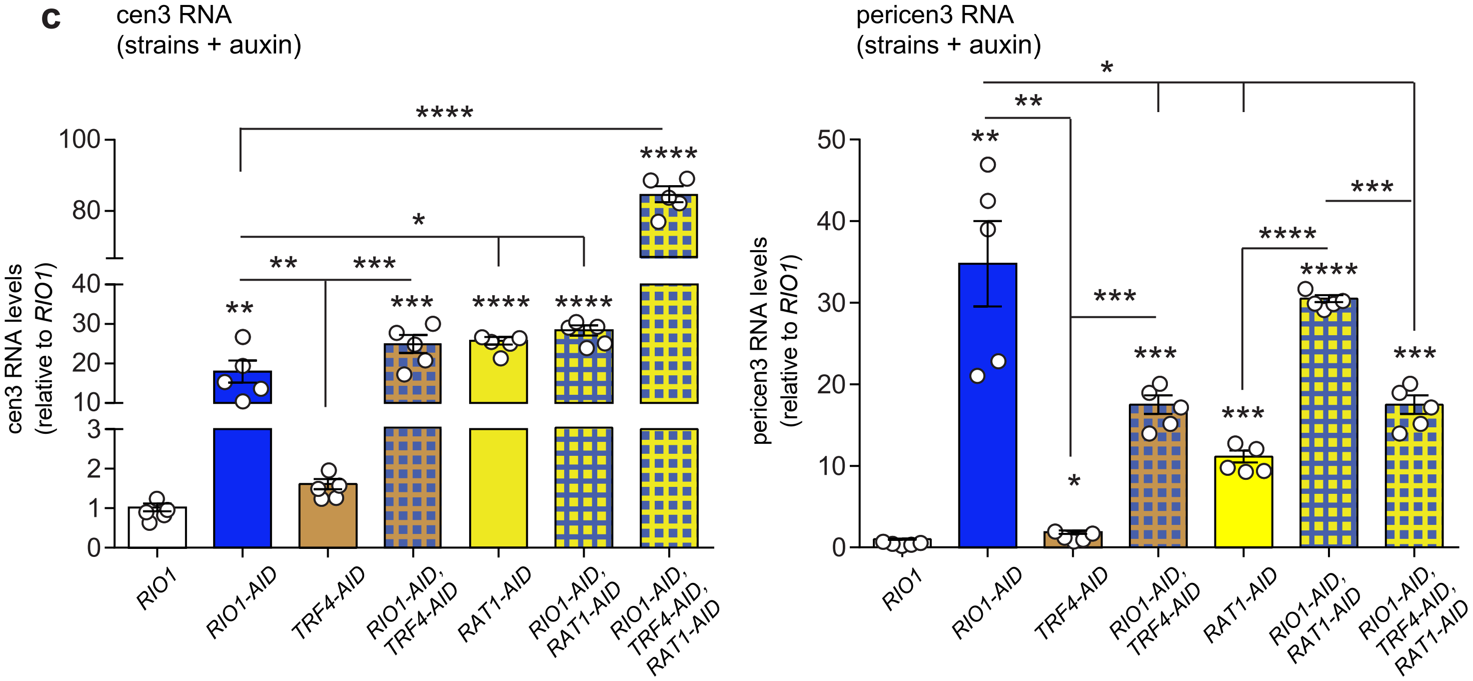

Supplementary Figure 4 (continued)

Supplementary Fig. 4 │ Regulation of cen- and pericenRNA levels by Rio1, Rat1, and TRAMP/nuclear exosome activities. a, Left: Trf4-AID and Rio1-AID protein levels (anti-AID) in the *TRF4-AID, RIO1-AID,* and *TRF4-AID RIO1-AID* strains treated for 1h with 500µM auxin or a mock, as revealed by western blot analysis. Pgk1 acted as the loading control. Right: Western blot of the Rat1-AID protein detected at time = 0 min, 20 min, and 60 min following 500µM auxin treatment (at time = 0 min). Pgk1 (anti-Pgk1) was probed as the loading control. **b**, Confidence levels (*P*-values) for the data plotted in Fig. 4a (obtained from five independent experiments, *n*=5) were calculated with the unpaired, two-tailed student t-test. **c**, Cen3 and pericen3 RNA levels quantitated by RT-qPCR analysis in the indicated strains, which were treated for 1h with 500µM auxin. All data were normalised to those of *ACT1* mRNA (quantitated by RT-qPCR analysis), and then referenced to the corresponding transcript levels measured in the *RIO1* strain (value = 1). The singular data (white circles) were obtained from five experimental replicates (*n*=5) and are shown combined as mean ± SEM. Confidence levels (*P*-values) were calculated with the unpaired, two-tailed student t-test.

Supplementary Figure 5

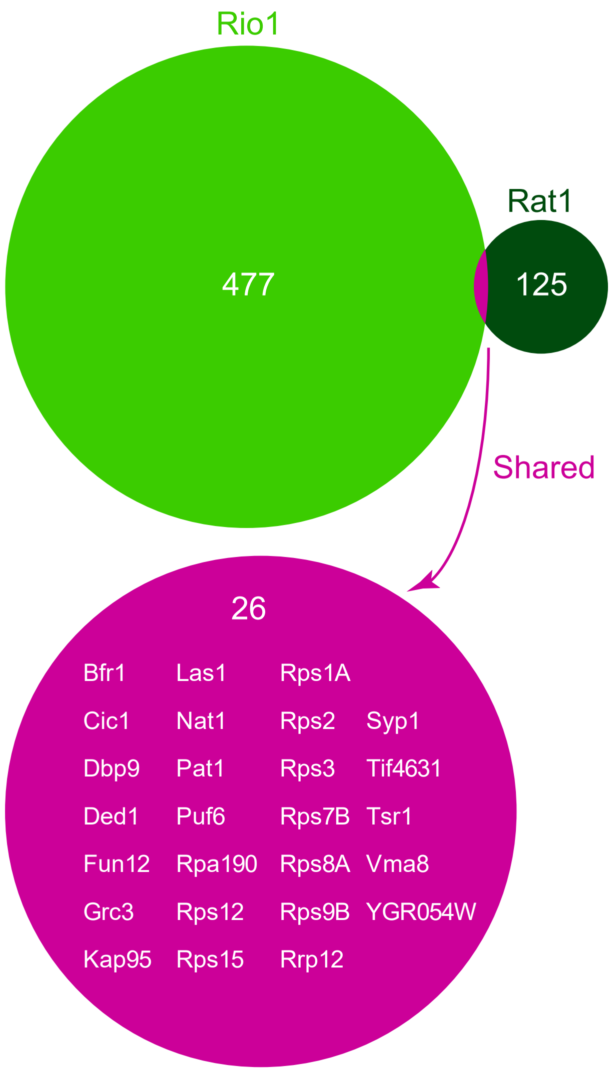

Supplementary Fig. 5 │ Overlap between the Rio1 and Rat1 interactomes. The number of proteins consistently co-purifying with Rio1 or Rat1 ($P \leq 0.05$) in three independent biological experiments ($n=3$). The individual proteins are listed in Supplementary Data 1. The pink circle indicates the proteins that co-purified with both Rio1 and Rat1.

Supplementary Figure 6

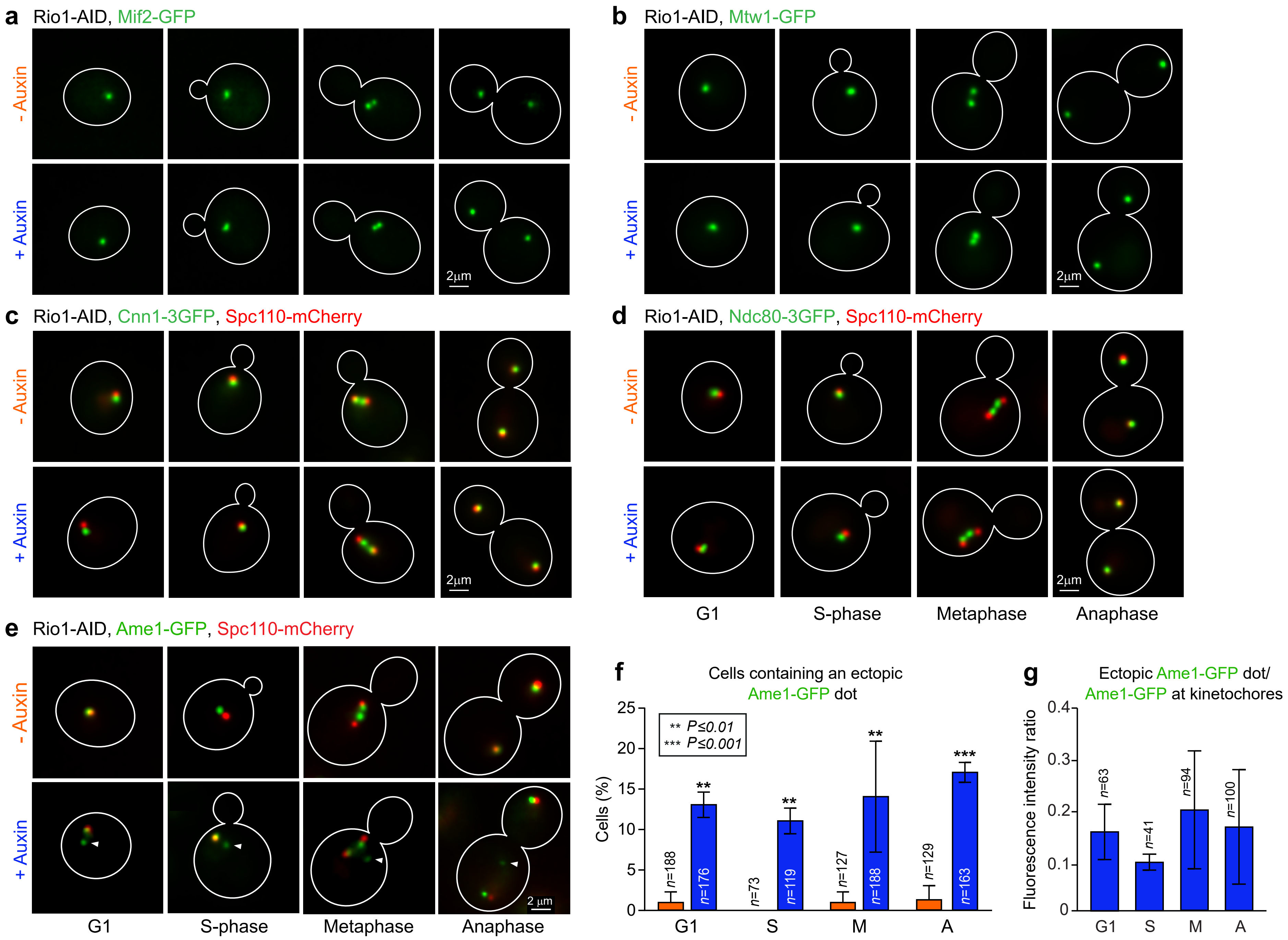

Supplementary Fig. 6 │ Localisation of kinetochore proteins in Rio1-depleted yeast. a-e, Representative images of GFP-labelled kinetochore reporter proteins occurring in different cell cycle stages (as revealed by budding state or position of spindle pole body protein Spc110-mCherry) of *RIO1-AID* strains treated for 1h with 500µM auxin or a mock (**a**, Mif2-GFP; **b**, Mtw1-GFP; **c**, Cnn1-3GFP; **d,** Ndc80-3GFP; **e**, Ame1-GFP). **f**, Percentage of cells containing an unaligned, ectopic Ame1-GFP signal (indicated by the white arrowheads) in different cell cycle stages (G1, S-phase (S), metaphase (M), and anaphase (A)). The number of analysed cells (*n*) deriving from three biological replicates are indicated. The data are shown as mean ± SD. *P*-values were calculated with the unpaired, two-tailed student t-test. **g**, Ratio between the fluorescence intensity of the unaligned, ectopic Ame1-GFP signal, and that of Ame1-GFP localising at spindle-bound kinetochores in the various cell cycle stages of *RIO1-AID* cells treated with 500µM auxin. The number of analysed cells (*n*) deriving from three biological replicates are indicated. The data are shown as mean ± SD. *P*-values were calculated with the unpaired, two-tailed student t-test.

Supplementary Figure 7

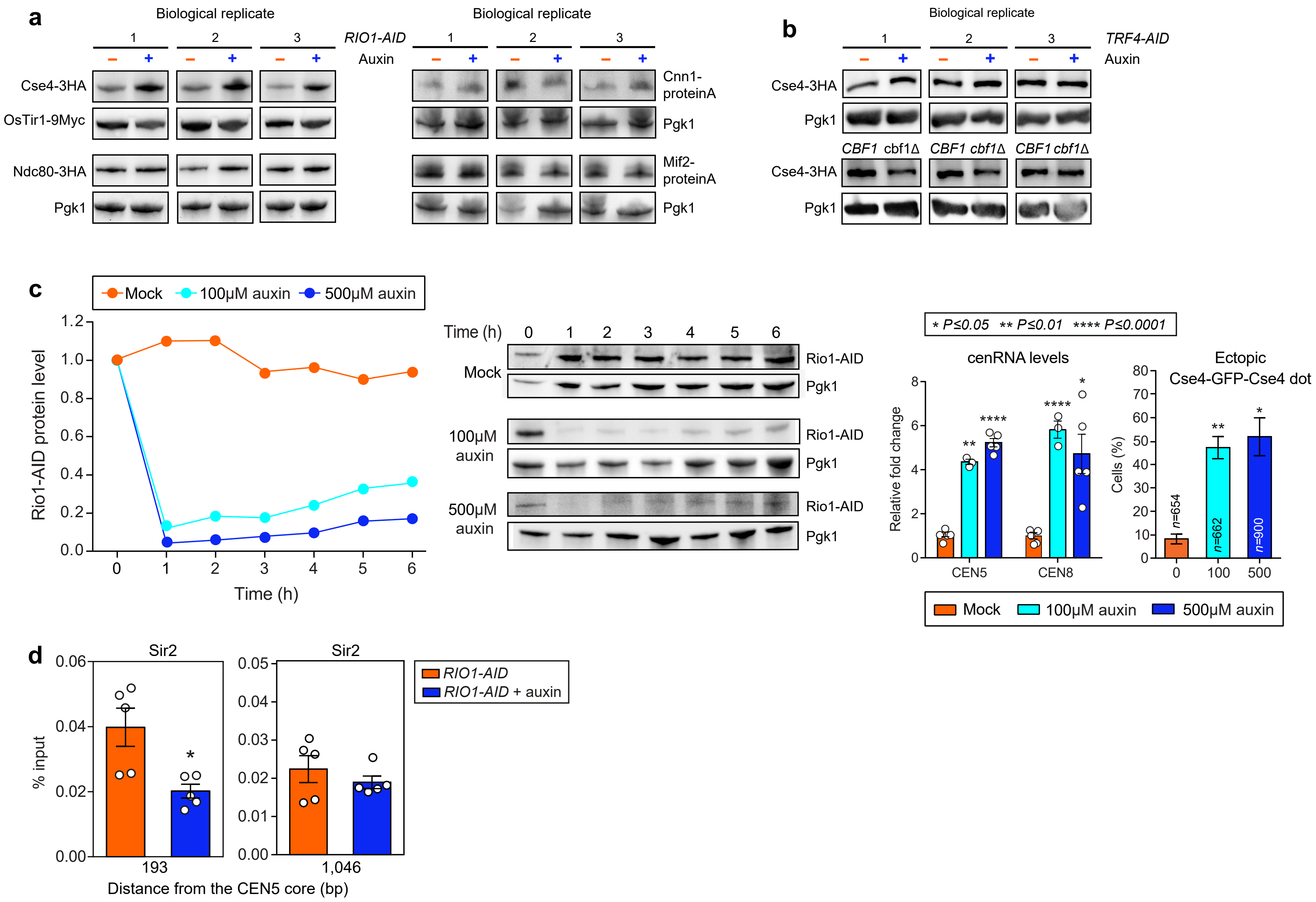

Supplementary Figure 7 (continued)

Supplementary Fig. 7 │ Analysis of kinetochore protein and cenRNA levels, and of histone deacetylase Sir2 bound at pericentromere regions in yeast containing or depleted of Rio1, Trf4 or Cbf1. a, Cse4-3HA (anti-HA), Ndc80-3HA (anti-HA), Cnn1-proteinA (peroxidase anti-peroxidase), and Mif2-proteinA (peroxidase anti-peroxidase) identified by western blot analysis of three independent *RIO1-AID* cultures (*n*=3) treated for 1h with 500µM auxin or a mock. OsTir1-9Myc (anti-Myc) or Pgk1 (anti-Pgk1) acted as the loading controls. Cse4-3HA, Mif2-proteinA, Cnn1-proteinA, and Ndc80-3HA levels were plotted in Fig. 5c. **b**, Cse4-3HA (anti-HA) identified by western bot analysis of three independent *TRF4-AID* cultures (*n*=3) treated for 1h with 500µM auxin or a mock, and of three wild-type (*CBF1*) and *cbf1Δ* yeast cultures (*n*=3 for each). Pgk1 (anti-Pgk1) acted as the loading control. Cse4-3HA levels were plotted in Fig. 7c. **c**, Rio1-AID protein levels in *RIO1-AID* cultures treated with a mock, 100µM or 500µM auxin for 6h (three replicates per condition, *n*=3). Rio1-AID protein levels (anti-AID) were measured, related to those quantitated for protein Pgk1 (anti-Pgk1; loading control), and then normalised to the value at time point 0h (value = 1). Cen5 and Cen8 RNA levels quantitated by RT-qPCR analysis of *RIO1-AID* cells treated for 1h with a mock, 100µM or 500µM auxin (five biological replicates per condition, *n*=5), and were normalised to those of *ACT1* mRNA (quantitated by RT-qPCR analysis), and then referenced to the corresponding transcript levels measured in the *RIO1* (wild-type) strain (value = 1). Error bars represent ± SEM. *RIO1-AID* cells (%) evidencing the ectopic Cse4-GFP-Cse4 dot phenotype when treated for 1h with a mock, 100µM or 500µM auxin (three replicates per condition, *n*=number of cells analysed per condition; error bars represent ± SD). **d**, Sir2 levels (as percentage of input) at periCEN5 (covering 60-193 bp and 936-1,046 bp downstream of CEN5) as measured by anti-Sir2 ChIP-qPCR analysis in *RIO1-AID* cells treated for 1h with 500µM auxin or a mock (five biological replicates per condition, *n*=5). Error bars represent ± SEM. *P*-values were calculated with the unpaired, two-tailed student t-test.

Supplementary Figure 8

a

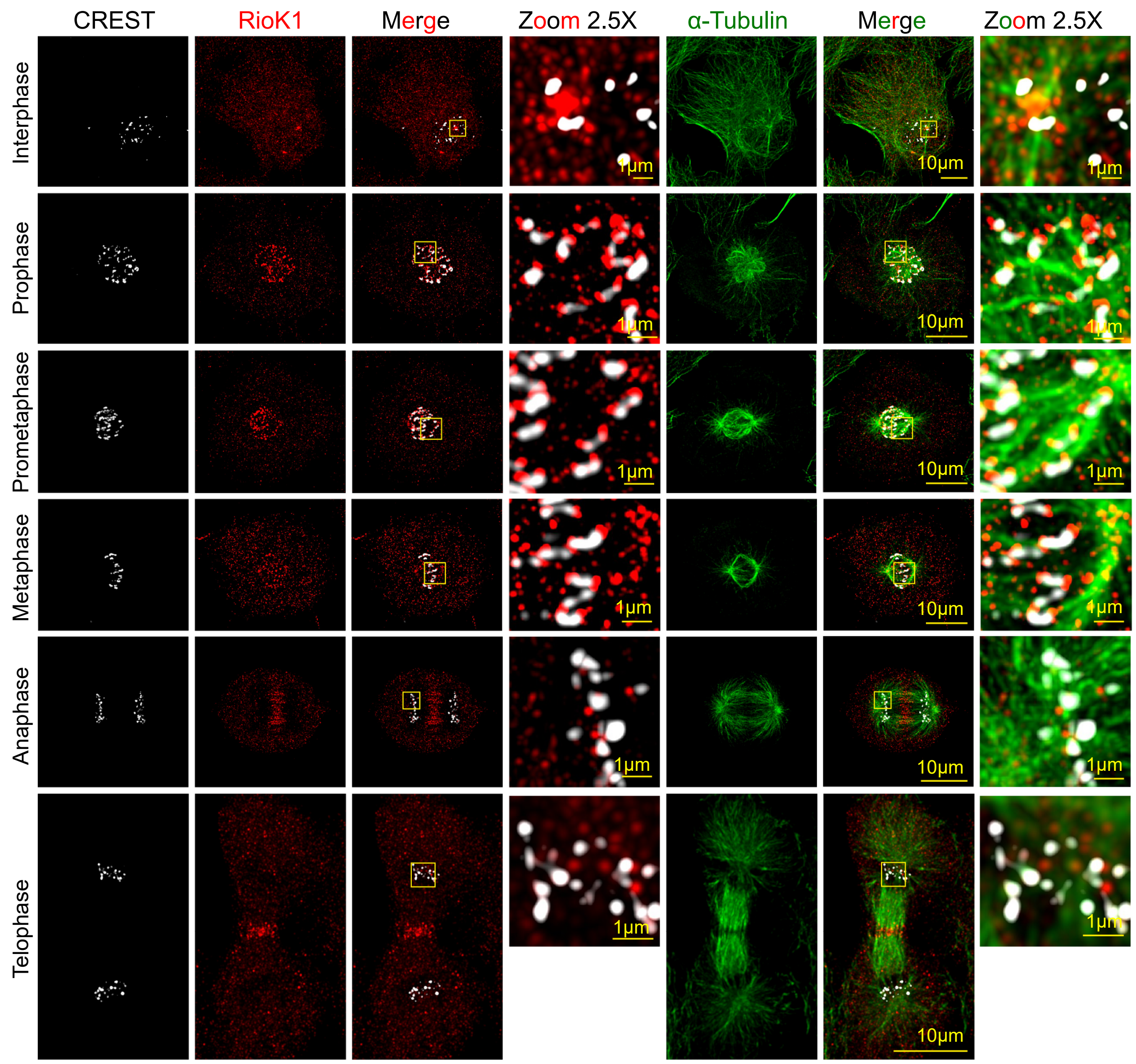

b **Scheme for cell enrichment in interphase, and subsequent analyses**

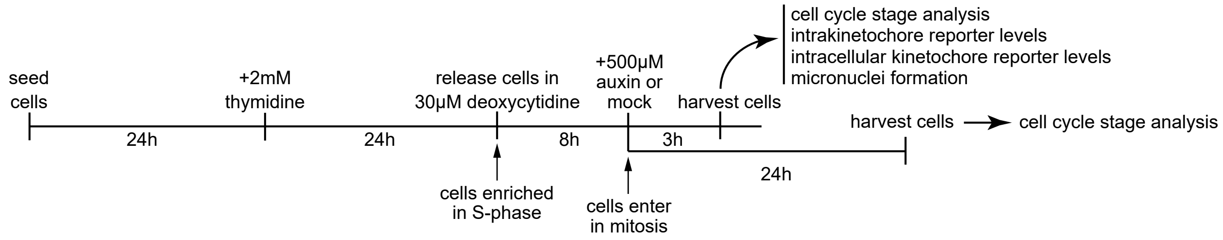

c

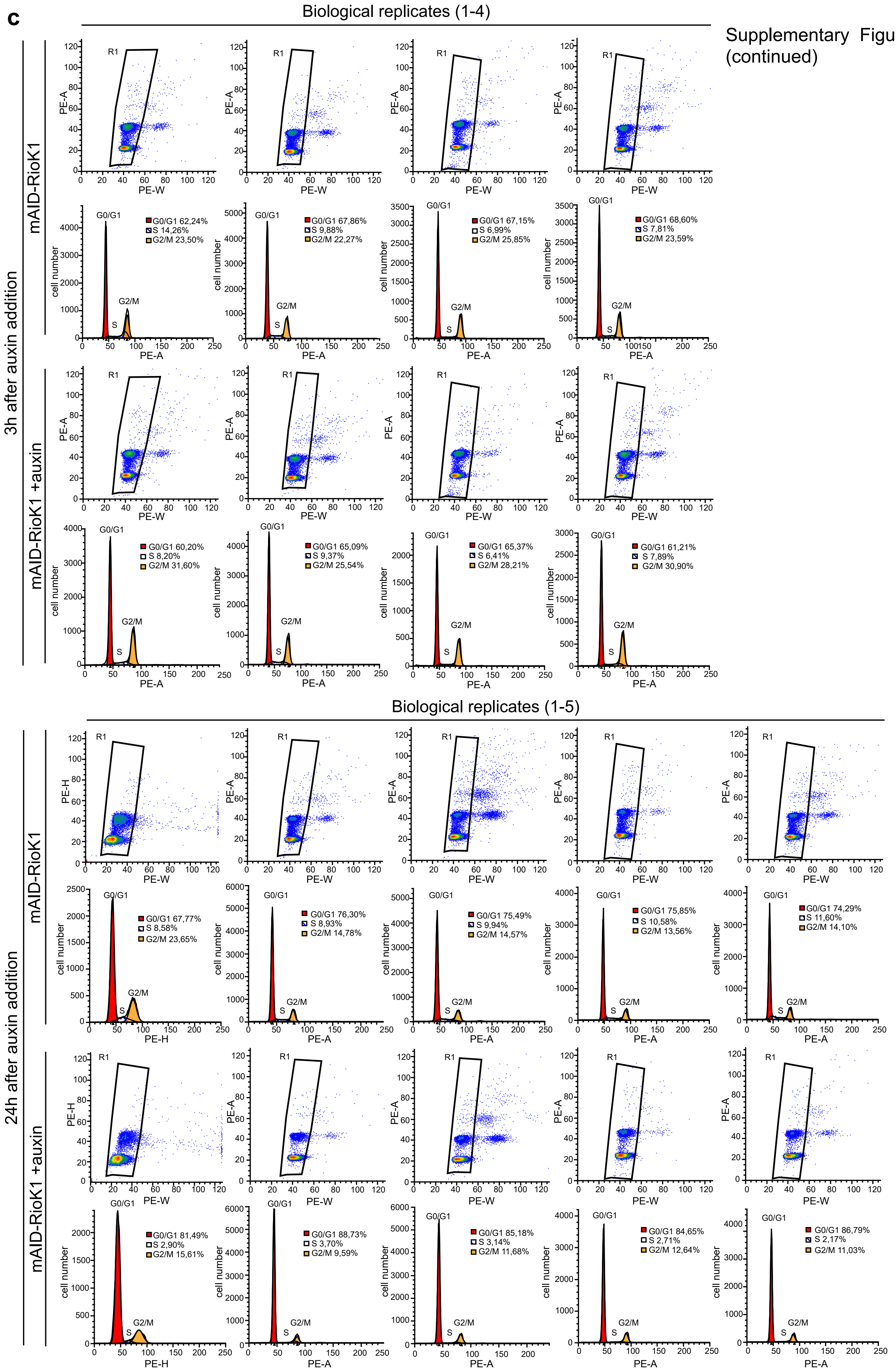

Supplementary Figure 8 (continued)

Supplementary Figure 8 (continued)

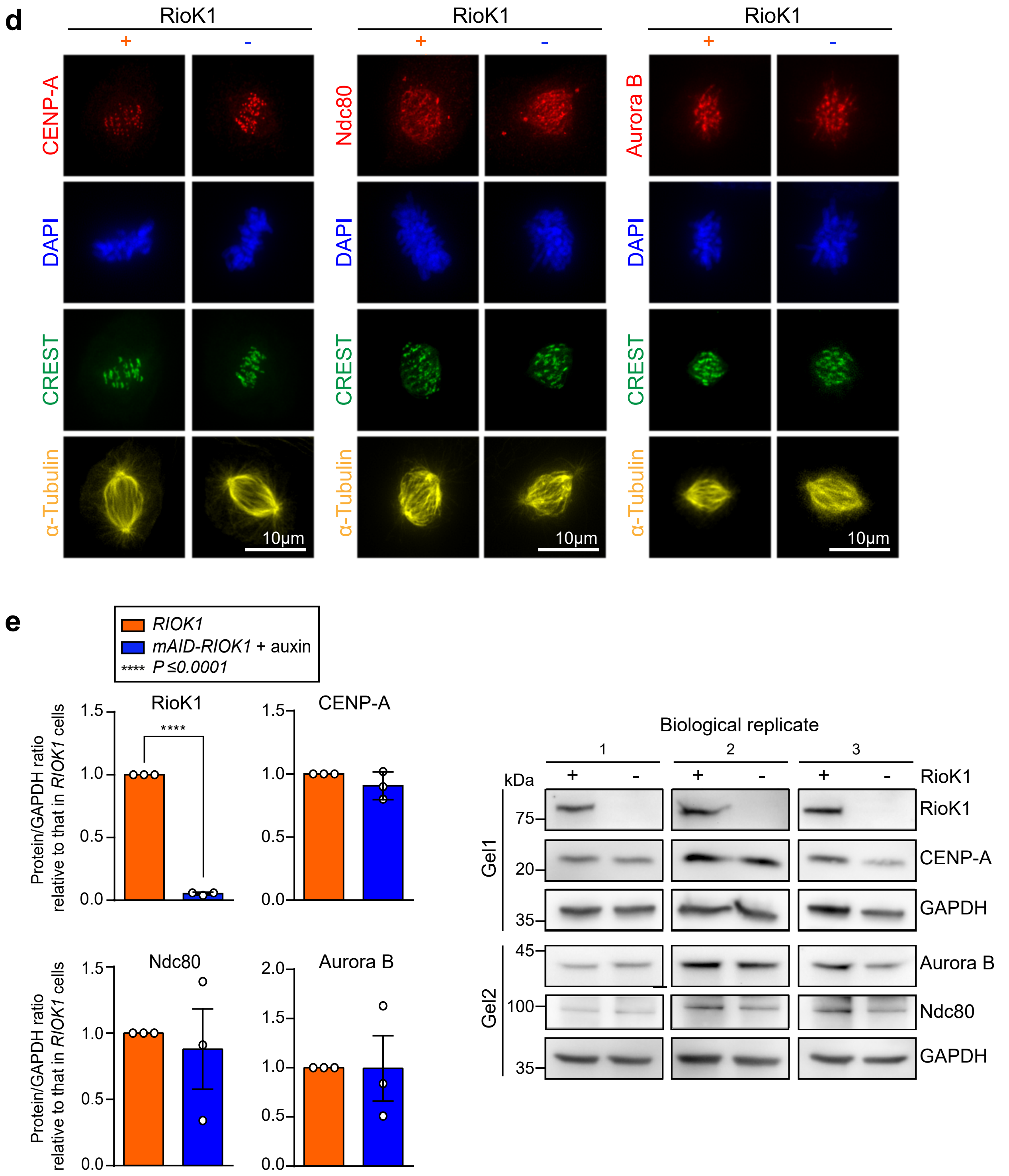

Supplementary Fig. 8 │ RioK1 promotes correct cenRNA levels, faithful kinetochore assembly, and chromosome stability in human cells. a, Representative immunofluorescence (IF) images of RioK1 in all cell cycle stages. Centromere-binding proteins (CREST) and α-tubulin mark the centromeres and microtubules, respectively. RioK1 levels measured at kinetochores through the cell cycle are plotted in Fig. 8a. **b**, Scheme to study *mAID-RIOK1* cells enriched in and released from interphase that enter mitosis in the presence of 500μM auxin or a mock. After 3h or 24h of growth in the presence of auxin or a mock, the cells were analysed for (i) cell cycle stage distribution (FACS; Fig. 8c, Supplementary Fig. 8c), (ii) kinetochore reporter protein levels at kinetochores (IF imaging; Fig. 8e, Supplementary Fig. 8d), and within cells (western blot; Supplementary Fig. 8e), and (iii) micronuclei formation (imaging following DAPI staining; Fig. 8f).

Supplementary Figure 8 (continued)

c, Plots of propidium iodide-based FACS analyses of *mAID-RIOK1* cells after 3h (upper panels) or 24h (bottom panels) of growth in the presence of auxin or a mock (corresponding to Fig. 8c). Biological replicates were *n*=4 (3h) or *n*=5 (24h). For each replicate, the PE-W vs PE-A or H plots and cell cycle profiles (cell number vs PE-A or H) with the percentage of cell cycle stages (G0/G1, S, G2/M) are shown. **d**, Representative IF images of kinetochore proteins CENP-A, Ndc80, and Aurora B in metaphase *mAID-RIOK1* cells following 3h of treatment with 500µM auxin or a mock at mitotic entry. Intrakinetochore levels of the three reporter proteins were measured and plotted in Fig. 8e. **e**, Whole-cell levels of kinetochore proteins CENP-A, Ndc80, and Aurora B in metaphase *mAID-RIOK1* cells following 3h of treatment with 500µM auxin or a mock at mitotic entry. Samples were taken from the same cultures as in Supplementary Fig. 8d, and were analysed by western blot (anti-CENP-A, anti-Ndc80, anti-Aurora B). Analysis of mAID-RioK1 (anti-RioK1) and GAPDH (anti-GAPDH) serving as controls for mAID-RioK1 depletion and sample loading, respectively. The data (white symbols) are shown as the means ± SEM of three biological replicates (*n*=3). Confidence levels (*P*-values) were calculated with the unpaired, two-tailed student t-test.

Supplementary Figure 9

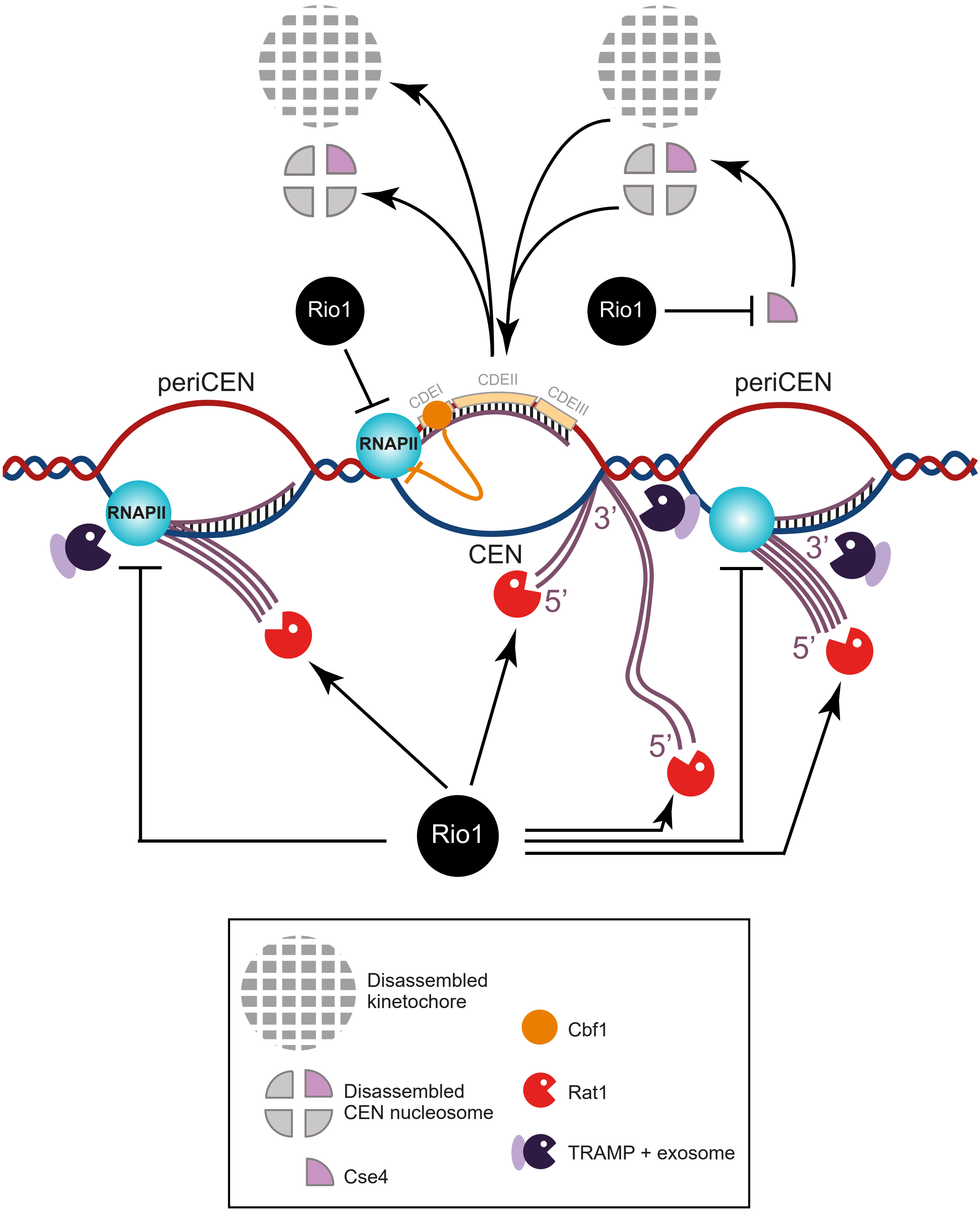

Supplementary Fig. 9 │ Working model of how yeast Rio1 promotes low cen- and noncoding pericenRNA levels in early S-phase. Following CEN and periCEN replication, Rio1 downregulates local RNAPII access and activity, and promotes cen- and noncoding pericenRNA turnover by 5’-3’ exoribonuclease Rat1, which occurs in parallel to cen- and noncoding pericenRNA degradation by the 3’-5’ TRAMP/nuclear exosome complex[1,2]. In addition, transcription factor Cbf1 acts as a road block for RNAPII[3]. Although Rio1 strongly downregulates noncoding pericenRNA levels, the latter occur in >two magnitude higher numbers than those deriving from the enclosed centromeres. Rio1 activity also ensures low levels of Cse4 and its correct deposition in centromeric nucleosomes following CEN replication.

Supplementary Figure 10

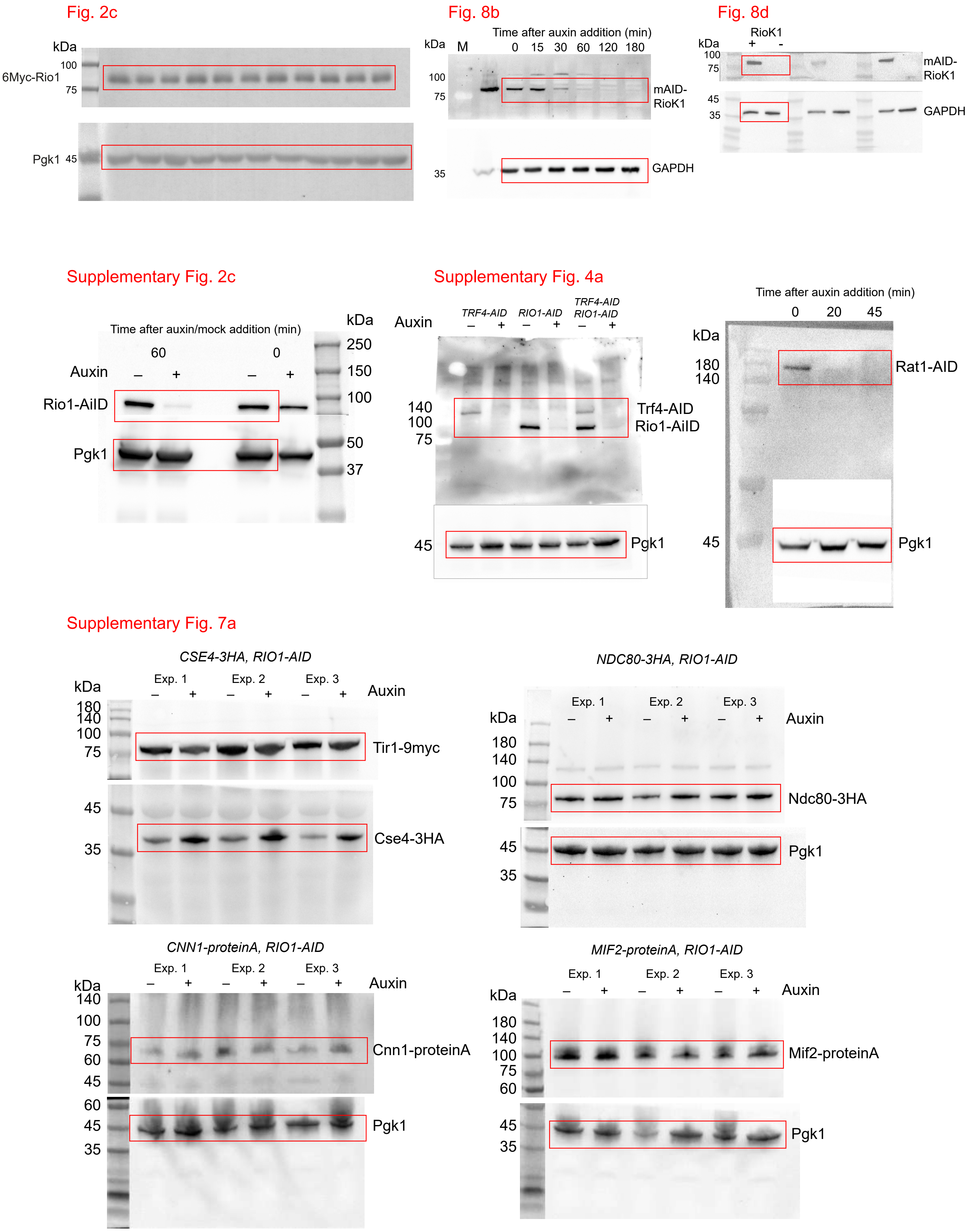

Supplementary Figure 10 (continued)

Supplementary Fig. 7b

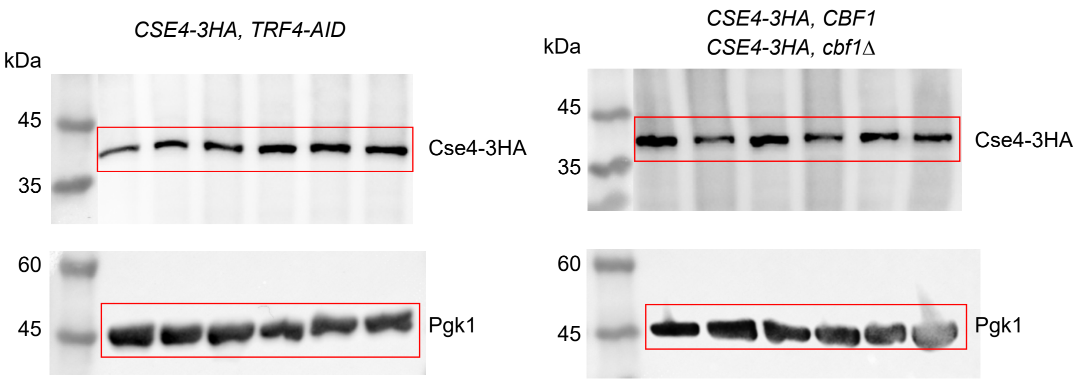

Supplementary Fig. 7c

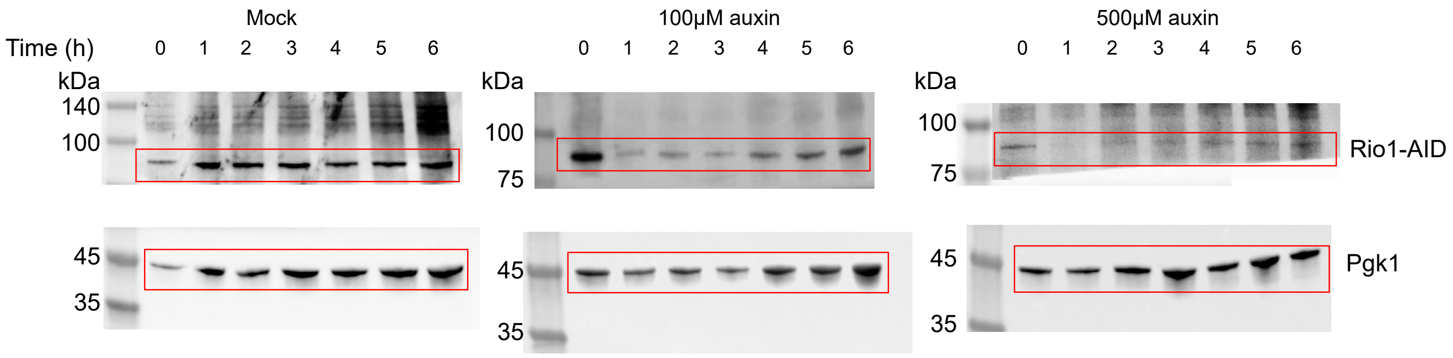

Supplementary Fig. 8e

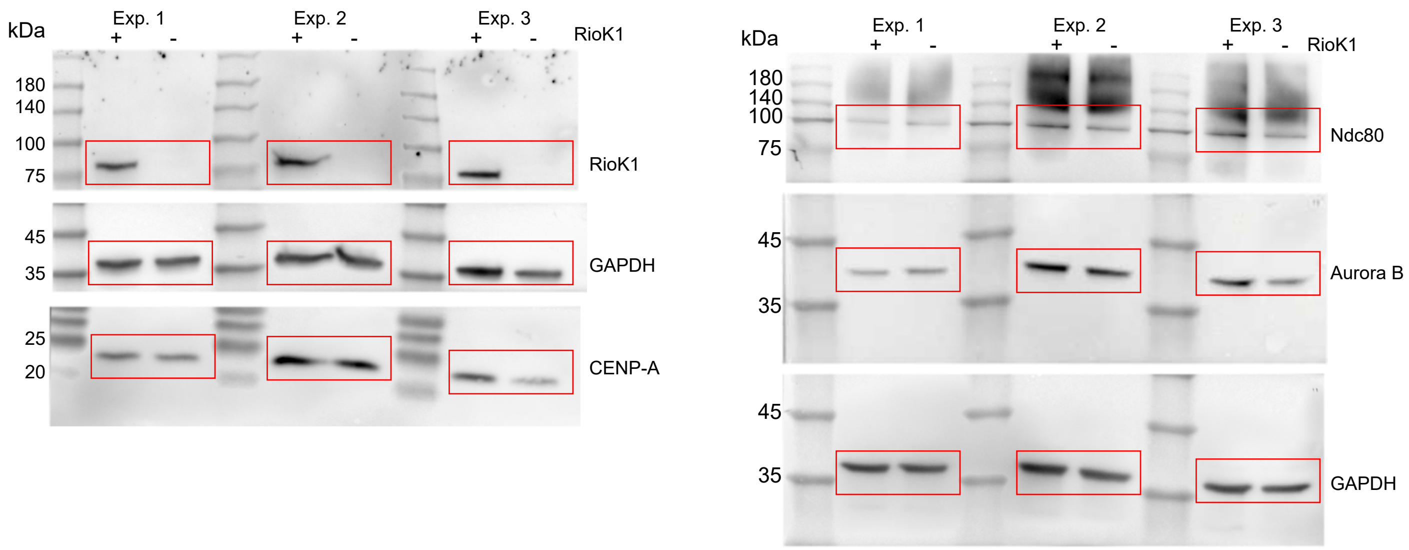

Supplementary Fig. 10 │ Full-size images of western blots, parts of which (indicated by the red rectangles) are shown in the named, main figures.

Supplementary Table 1. Yeast strains used in this study.

All strains derived from *S. cerevisiae* W303-1A.

Name	Genotype	Origin
PDW001	*MATa, ade2-1 trp1-1 can1-100 his3-11,15 leu2-3,112 ura3-1*	K. Nasmyth
PDW2798	*MATa, RIO1-AID::KanMX4,* P_{ADH1}*-OsTIR1-9myc::URA3*	Ref[4]
PDW2380	*MATa, 6Myc-RIO1, NDC80-GFP::URA3*	Ref[5]
PDW3086	*MATa, TUB1-GFP::URA3*	This study
PDW3104	*MATa, TUB1-GFP::URA3, ndc10-1*	This study
PDW2302	P_{ADH1}*-OsTIR1-9myc::URA3*	Ref[6]
PDW3071	*MATa, cbf1::HIS3MX6*	This study
PDW3073	*MATa, RIO1-AID::KanMX4,* P_{ADH1}*::OsTIR1-9myc::URA3, cbf1::HIS3MX6*	This study
PDW3162	*MATa, TRF4-AID::KanMX4,* P_{ADH1}*-OsTIR1-9myc::URA3*	This study
PDW3161	*MATa, RIO1-AID::KanMX4, TRF4-AID::KanMX4,* P_{ADH1}*-OsTIR1-9myc::URA3*	This study
PDW3199	*MATa, RAT1-AID::KanMX4,* P_{ADH1}*-OsTIR1-9myc::URA3*	This study
PDW3216	*MATa, RIO1-AID::KanMX4, RAT1-AID::KanMX4,* P_{ADH1}*-OsTIR1-9myc::URA3*	This study
PDW3221	*MATa, RIO1-AID::KanMX4, RAT1-AID::KanMX4, TRF4-AID::KanMX4,* P_{ADH1}*-OsTIR1-9myc::URA3*	This study
PDW3234	*MATa, pP*$_{GAL}$*-RIO1::URA3, pep4::KanMX4*	This study
PDW3235	*MATa, pP*$_{GAL}$*-ProteinA-TEV-Flag-RIO1::URA3, pep4::KanMX4*	This study
PDW3222	*MATa, RAT1-TEV-ProteinA::KanMX4*	This study
PDW3002	*MATa, RIO1-AID::KanMX4,* P_{ADH1}*::OsTIR1-9myc::URA3, NDC80-3GFP::KanMX4, SPC110-mCherry::HphMX3*	This study
PDW3063	*MATa, RIO1-AID::KanMX4,* P_{ADH1}*-OsTIR1-9myc::URA3, CSE4-GFP-CSE4, SPC110-mCherry::HphMX3*	This study
PDW2974	*MATa, RIO1-AID::KanMX4,* P_{ADH1}*::OsTIR1-9myc::URA3, MIF2-GFP::HIS3MX6*	This study
PDW2975	*MATa, RIO1-AID::KanMX4,* P_{ADH1}*::OsTIR1-9myc::URA3, CNN1-3GFP::KanMX4, SPC110-mCherry::HphMX3*	This study
PDW3083	*MATa, RIO1-AID::KanMX4,* P_{ADH1}*::OsTIR1-9myc::URA3, MTW1-GFP::HIS3MX6*	This study
PDW3098	*MATa, RIO1-AID::KanMX4,* P_{ADH1}*::OsTIR1-9myc::URA3, AME1-GFP::HIS3MX6, SPC110-mCherry::HphMX3*	This study

PDW3266	*MATa, RIO1-AID::KanMX4, P_{ADH1}::OsTIR1-9myc::URA3, CSE4-3HA::TRP1*	This study
PDW3279	*MATa, RIO1-AID::KanMX4, P_{ADH1}::OsTIR1-9myc::URA3 MIF2-TEV-ProteinA::KanMX6*	This study
PDW3278	*MATa, RIO1-AID::KanMX4, P_{ADH1}::OsTIR1-9myc::URA3, CNN1-TEV-ProtenA::KanMX6*	This study
PDW3269	*MATa, RIO1-AID::KanMX4, P_{ADH1}::OsTIR1-9myc::URA3, NDC80-3HA::TRP1*	This study
PDW3145	*MATa, RIO1-AID::KanMX4, P_{ADH1}::OsTIR1-9myc::URA3, CSE4-GFP-CSE4, SPC110-mCherry::HphMX3, TUB1-mCherry::TRP1*	This study
PDW3097	*MATa, RIO1-AID::KanMX4, P_{ADH1}::OsTIR1-9myc::URA3, CSE4-GFP-CSE4, SPC110-mCherry::HphMX3, NOP1-mCherry::LEU2*	This study
PDW3250	*MATa, RIO1-AID::KanMX4, P_{ADH1}::OsTIR-9myc::URA3, CSE4-GFP-CSE4, SPC110-mCherry::HphMX3, RAP1-CFP::URA3*	This study
PDW3189	*MATa, RIO1-AID::KanMX4, P_{ADH1}::OsTIR1-9myc::URA3, CSE4-GFP-CSE4*	This study
PDW3124	*MATa, OsTIR1-9myc::URA3, ade2-101, CFIII (CEN3.L.YPH278) SUP11-1 URA3*	This study
PDW3116	*MATa, RIO1-AID::KanMX4, OsTIR1-9myc::URA3, ade2-101, CFIII (CEN3.L.YPH278) SUP11-1 URA3*	This study
PDW3077	*MATa, RIO1-AID::KanMX4, P_{ADH1}-OsTIR1-9myc::URA3, CSE4-GFP-CSE4, SPC110-mCherry::HphMX3, cbf1::HIS3MX6*	This study
PDW3260	*MATa, TRF4-AID::KanMX4, P_{ADH1}-OsTIR1-9myc::URA3, CSE4-GFP-CSE4, SPC110-mCherry::HphMX3*	This study
PDW3290	*MATa, TRF4-AID::KanMX4, P_{ADH1}-OsTIR1-9myc::URA3, CSE4-3HA::TRP1*	This study
PDW3291	*MATa, cbf1::HIS3MX6, CSE4-3HA::TRP1*	This study

Supplementary Table 2. Oligomers used for qPCR analysis in this study.

Name	Sequence (5’-3’)	Direction	Target
**Yeast**			
CIBIOL25	ATAGAGGTGCAGATTGTTGCG	Forward	Cen13 long RNA
CIBIOL26	GTCTGAGTAGGTCTCGATCTCG	Reverse	
CIBIOL29	GGCATTGTCATAGATGTGGAAGA	Forward	Cen7 long RNA
CIBIOL30	GATAAGAGATTCGAGTTGTTAGCTGG	Reverse	
CIBIOL31	TCAGCCTCCGAAGGGAGTTGTA	Forward	Cen2 long RNA
CIBIOL32	GGCGTGCCAGTTCCACTAAATT	Reverse	
CIBIOL35	TGTAACATTAGGGTTCTGTTACTTGTC	Forward	Cen2 long RNA
CIBIOL36	GTAATAAGGTTGATGTTGCAAGAGGTA	Reverse	
CIBIOL37	GCAATTACGGATACGGTTGGTC	Forward	Cen11 long RNA
CIBIOL38	AGAGAAGCGATTTCTAATCAGTTCAT	Reverse	
CIBIOL41	TGTCCGCTTTCGTGCACAAG	Forward	Cen4 long RNA
CIBIOL42	TGGTTGGGACTCCTATATGATGGC	Reverse	
CIBIOL43	GATCTCCTAATTGCTCCTCGAC	Forward	Cen14 long RNA
CIBIOL44	GACTGGTGAAATACCTACTGACG	Reverse	
CIBIOL47	CTTTTCCTTCACTGCATTCCGG	Forward	Cen9 long RNA
CIBIOL48	ACCCAAAGACGGGTAAACCATTA	Reverse	
CIBIOL51	AAGATTGCTCAACGTGAAAGACG	Forward	Cen8 long RNA
CIBIOL52	GTGTTTATTTTAGCAGTTACCTCGAG	Reverse	
CIBIOL161	GCTTGCAGCGTAGCTAAACTCT	Forward	periCEN3, upstream
CIBIOL162	TTCCATATTGTTTGGCGCTGA	Reverse	
CIBIOL189	GCGGCAAATAGTACAAATAAGTCACAT	Forward	CEN3
CIBIOL190	GCCTGATTTCTTTTTTAACTTTCGGAA	Reverse	
CIBIOL139	TTCGTCCTTAATGTCCGCGA	Forward	periCEN3, downstream
CIBIOL140	GGGGTATATGAATGGGGCGG	Reverse	
CIBIOL151	CTACATGCTACATAAGTCCGAGA	Forward	periCEN5, upstream
CIBIOL152	GCACGTGATTAATTTGATGTTTCA	Reverse	
CIBIOL201	CCCAATTTAATTTCATTTTCTATTTC	Forward	CEN5
CIBIOL202	GGGTTCTTTTCGGAAATCTAATACTG	Reverse	
CIBIOL143	CCACTGTTGGCGTTTCAACT	Forward	periCEN5, downstream
CIBIOL144	TATGTGCGGCTTTGTCAGCA	Reverse	
CIBIOL159	CGTTTTGTGGATGTGTAATTGTTGG	Forward	periCEN8, upstream
CIBIOL160	TCATTGGTGCATTACATTCCTTACC	Reverse	
CIBIOL153	AGTTCGGAACACAAAACCCAATGT	Forward	CEN8
CIBIOL154	CTCCAACAATTACACATCCACAAAACG	Reverse	
CIBIOL112	ACCATTCGACGCAAGGAACA	Forward	periCEN8, downstream
CIBIOL113	AAGCCCAAATCTCTTGCCGA	Reverse	

CIBIOL114	TGCAAACCGCTGCTCAATCT	Forward	*ACT1*
CIBIOL115	ACCGGCAGATTCCAAACCCA	Reverse	
CIBIOL378	GCCAGTAGCGACACCACACA	Forward	Centromere like region 1 (Chr3)[7]
CIBIOL379	CCCAGACTTCAGGCGCTGTT	Reverse	
CIBIOL380	ACGCATGCTTCGTAAGGCGA	Forward	Centromere like region 17 (Chr13)[7]
CIBIOL381	TGGCGTCGTTTACTGCCGTT	Reverse	
CIBIOL382	GCCTAGGCCGCTGCTACTTT	Forward	Centromere like region 18 (Chr10)[7]
CIBIOL383	CAGAGACGCGTGGAAGCACC	Reverse	
CIBIOL384	GCCTGTGGGAATACTGCCAG	Forward	rDNA (RDN25)[8]
CIBIOL385	CCATCTTTCGGGTCCCAACAGC	Reverse	
CIBIOL386	TCCTAGGTTATCTCATCGGTACT	Forward	SLP1 Promoter[8]
CIBIOL387	ACTATATCCATTGCGTCCTTTCT	Reverse	
CIBIOL388	ACAGCACAACACGCTTACCA	Forward	SAP4 Promoter[8]
CIBIOL389	CCAGCCCTAAATCCCCTAAA	Reverse	
**Human**			
CIBIOL343	CTTCCTTCGAAACGGGTATATCT	Forward	D11Z1-CEN
CIBIOL344	GCTCCATCAGCAGGATTGT	Reverse	
CIBIOL209	TGGATCCCAGAACAAGAATGAT	Forward	*RIOK1*
CIBIOL211	TCCCGATCTTTGAACACCAA	Reverse	
CIBIOL197	CAACGGATTTGGTCGTATTGG	Forward	*GAPDH*
CIBIOL198	GCAACAATATCCACTTTACCAGAGTTAA	Reverse	

Supplementary references

1. Ling, Y.H. & Yuen, K.W.Y. Point centromere activity requires an optimal level of centromeric noncoding RNA. *Proc Natl Acad Sci U S A* **116**, 6270-6279 (2019).

2. Houseley, J., Kotovic, K., El Hage, A. & Tollervey, D. Trf4 targets ncRNAs from telomeric and rDNA spacer regions and functions in rDNA copy number control. *The EMBO journal* **26**, 4996-5006 (2007).

3. Hedouin, S., Logsdon, G.A., Underwood, J.G. & Biggins, S. A transcriptional roadblock protects yeast centromeres. *Nucleic Acids Research* **50**, 7801-7815 (2022).

4. Iacovella, M.G. *et al.* Integrating Rio1 activities discloses its nutrient-activated network in *Saccharomyces cerevisiae*. *Nucleic Acids Res* **46**, 7586-7611 (2018).

5. Iacovella, M.G. *et al.* Rio1 promotes rDNA stability and downregulates RNA polymerase I to ensure rDNA segregation. *Nature communications* **6**, 6643 (2015).

6. Nishimura, K., Fukagawa, T., Takisawa, H., Kakimoto, T. & Kanemaki, M. An auxin-based degron system for the rapid depletion of proteins in nonplant cells. *Nat Methods* **6**, 917-22 (2009).

7. Bobkov, G.O.M. *et al.* Spt6 is a maintenance factor for centromeric CENP-A. *Nature Communications* **11**(2020).

8. Hildebrand, E.M. & Biggins, S. Regulation of Budding Yeast CENP-A levels Prevents Misincorporation at Promoter Nucleosomes and Transcriptional Defects. *PLoS Genet* **12**, e1005930 (2016).
