# Supplementary material for: Rio1 downregulates centromeric RNA levels to promote the timely assembly of structurally fit kinetochores
Source: Nat Commun. 2023 Jun 1;14:3172. doi: 10.1038/s41467-023-38920-9 (PMC10235086; doi:10.1038/s41467-023-38920-9)
Supplement: Supplementary file 2 — Description of Additional Supplementary Files [file 41467_2023_38920_MOESM2_ESM.pdf]

Description of Additional Supplementary Files

File name: Supplementary Data 1
Description: Mass spectrometry data for Rio1 and Rat1 purifications (deriving from three biological replicates each).

File name: Supplementary Data 2
Description: Differentially expressed genes (annotated ORFs) identified in three independent experiments (*RIO1-AID* + auxin/*RIO1-AID* + mock). The applied filters were *P* adj <= 0.05 and Log2FoldChange <= -1 and >= +1.
