# Supplementary material for: Rio1 downregulates centromeric RNA levels to promote the timely assembly of structurally fit kinetochores
Source: Nat Commun. 2023 Jun 1;14:3172. doi: 10.1038/s41467-023-38920-9 (PMC10235086; doi:10.1038/s41467-023-38920-9)
Supplement: Supplementary file 5 — Reporting Summary [file 41467_2023_38920_MOESM5_ESM.pdf]

nature portfolio

Corresponding author(s): Peter De Wulf

Last updated by author(s): 20/04/2023

Reporting Summary

Nature Portfolio wishes to improve the reproducibility of the work that we publish. This form provides structure for consistency and transparency in reporting. For further information on Nature Portfolio policies, see our Editorial Policies and the Editorial Policy Checklist.

Statistics

For all statistical analyses, confirm that the following items are present in the figure legend, table legend, main text, or Methods section.

n/a	Confirmed	
☐	☒	The exact sample size (*n*) for each experimental group/condition, given as a discrete number and unit of measurement
☐	☒	A statement on whether measurements were taken from distinct samples or whether the same sample was measured repeatedly
☐	☒	The statistical test(s) used AND whether they are one- or two-sided<br>*Only common tests should be described solely by name; describe more complex techniques in the Methods section.*
☐	☒	A description of all covariates tested
☐	☒	A description of any assumptions or corrections, such as tests of normality and adjustment for multiple comparisons
☐	☒	A full description of the statistical parameters including central tendency (e.g. means) or other basic estimates (e.g. regression coefficient) AND variation (e.g. standard deviation) or associated estimates of uncertainty (e.g. confidence intervals)
☐	☒	For null hypothesis testing, the test statistic (e.g. *F*, *t*, *r*) with confidence intervals, effect sizes, degrees of freedom and *P* value noted<br>*Give P values as exact values whenever suitable.*
☒	☐	For Bayesian analysis, information on the choice of priors and Markov chain Monte Carlo settings
☒	☐	For hierarchical and complex designs, identification of the appropriate level for tests and full reporting of outcomes
☒	☐	Estimates of effect sizes (e.g. Cohen's *d*, Pearson's *r*), indicating how they were calculated

Our web collection on statistics for biologists contains articles on many of the points above.

Software and code

Policy information about availability of computer code

Data collection	No software was used for data collection.
Data analysis	Analysis of our Illumina NovaSeq6000 RNA-Seq data (+ use of UMIs) was performed with Trimmomatic V.032 (Bolger, A.M., Lohse, M. & Usadel, B. Trimmomatic: a flexible trimmer for Illumina sequence data. Bioinformatics 30, 2114-20 (2014)), STAR (Dobin, A. et al. STAR: ultrafast universal RNA-seq aligner. Bioinformatics 29, 15-21 (2013)), BEDTools (Quinlan, A.R. & Hall, I.M. BEDTools: a flexible suite of utilities for comparing genomic features. Bioinformatics 26, 841-2 (2010), and R script (https://www.r-project.org/).<br>Our proteomics data were analyzed using R-script (https://www.r-project.org/) and the resulting interactome map created with Cytoscape 3.8.2 (Shannon, P. Cytoscape: A Software Environment for Integrated Models of Biomolecular Interaction Networks. Genome Research 13, 2498-2504 (2003)). FACS data were analysed using ModFit LT 4.0 software (Verity Software House). Statistical data analyses were performed with GraphPad Prism 6.01 (GraphPad Pro). Microscopy files were analysed with ImageJ (FIJI) v2.3.0 (National Institutes of Health, USA).

For manuscripts utilizing custom algorithms or software that are central to the research but not yet described in published literature, software must be made available to editors and reviewers. We strongly encourage code deposition in a community repository (e.g. GitHub). See the Nature Portfolio guidelines for submitting code & software for further information.

Data

Policy information about availability of data

All manuscripts must include a data availability statement. This statement should provide the following information, where applicable:

- Accession codes, unique identifiers, or web links for publicly available datasets
- A description of any restrictions on data availability
- For clinical datasets or third party data, please ensure that the statement adheres to our policy

All Source Data are provided in Supplementary Table 5. Filtered RNA reads were aligned to the sacCer3 genome (assembly R64). The RNA-Seq data are available via the Gene Expression Omnibus (GEO, NCBI) with accession number GSE218602. The proteomic data are available via ProteomeXchange with identifier number PXD038337. We have confirmed that all our data submitted to public repositories are indeed publically available.

Field-specific reporting

Please select the one below that is the best fit for your research. If you are not sure, read the appropriate sections before making your selection.

☒ Life sciences ☐ Behavioural & social sciences ☐ Ecological, evolutionary & environmental sciences

For a reference copy of the document with all sections, see nature.com/documents/nr-reporting-summary-flat.pdf

Life sciences study design

All studies must disclose on these points even when the disclosure is negative.

Sample size	For each experiment, samples sizes were defined by a specific "n" value. Then value either represented the number of experimental replicates that were performed (e.g., for western blots, RT-qPCR analyses, RNA-Seq experiments, cell cycle studies) or n represented the numbers of cells that were analysed deriving from a specifically (and indicated) number of experimental replicates (e.g., microscopy analyses). The legends to the Figures and supplementary figures always list th en value. The n-data themselves are found in Data Source Supplementary File 5.
Data exclusions	No data were ever excluded from our analyses.
Replication	To ensure the reproducibility of our data, "n" experimental replicates ranged from 3 to 7, as indicated in the text. As for the numner of "n" cells analysed (imaging) the n number ranged from 20-3000, as specifically indicated in the manuscrupt. All experiments were performed by different people in the lab to ensure replicability of results. Rigorous statistical analyses (student t-tests) were performed to reveal levels of confidence (P-values) of the reported findings. All attempts at replication were succesful.
Randomization	At least three yeast and human cell cultures (the number of cultures/experiments are indicated in the text) were grown independently (deven on different days) and then harvested under the same experimental conditions, hence allowing for independent data collection and analysis. Also, the experiments were repeated many times by different scientists, therewith eliminating person-dependent techical effects.
Blinding	Blinded group allocation was not releavant in our study as our experiments included both positive and negative controls.

Reporting for specific materials, systems and methods

We require information from authors about some types of materials, experimental systems and methods used in many studies. Here, indicate whether each material, system or method listed is relevant to your study. If you are not sure if a list item applies to your research, read the appropriate section before selecting a response.

Materials & experimental systems

n/a	Involved in the study	
☐	☒	Antibodies
☐	☒	Eukaryotic cell lines
☒	☐	Palaeontology and archaeology
☒	☐	Animals and other organisms
☒	☐	Human research participants
☒	☐	Clinical data
☒	☐	Dual use research of concern

Methods

n/a	Involved in the study	
☒	☐	ChIP-seq
☐	☒	Flow cytometry
☒	☐	MRI-based neuroimaging

Antibodies

Antibodies used	In the next paragraph (as in the Methods section in the manuscript) , we specific the antibodies (primary and secondary) used, the dilutions used for each for in immunofluorescence or western blot studies. Supplier names, antibody clone name and nr., and

catalogue nrs. are provided, as applicable both below and in the Methods section of the manuscript.

Anti-RNA polymerase II (mouse monoclonal, clone CTD4H8, Sigma Cat # 05-623), Living Colors full-length anti-GFP (polyclonal rabbit, Takara Cat # 632592), mouse monoclonal anti-Pgk1 (1:5,000; Life Technologies, Cat # 459250), mouse monoclonal anti-Myc 9E10 (1:1,000; Covance, Cat # MMS-150R). Horseradish peroxidase-conjugated goat anti-mouse (1:10,000; Bio-Rad, Cat # 170-6516), mouse polyclonal anti-RioK1 antibody (1:500 dilution; Abnova, Cat # H00083732-B02P), rabbit polyclonal anti-RioK1 antibody (1: 500 dilution; Novus, Cat # NBP2-473021), rabbit monoclonal GAPDH antibody (clone 14C10, Cell Signaling, Cat # 2118), mouse monoclonal anti-alpha-tubulin (clone B512; Sigma, Cat # T5168), polyclonal horseradish peroxidase-conjugated anti-mouse IgG (1:5000 dilution; Dako, Cat # P0161), polyclonal horseradish peroxidase-conjugated anti-rabbit IgG (1:5000 dilution; Dako, Cat # P0448), goat polyclonal anti-Sir2 (Santa Cruz, Cat # sc-6666), anti-Aurora B (rabbit polyclonal, 1:500, Abcam, Cat # ab2254), anti-CENP-A (mouse monoclonal, 1:500,ThermoFisher Scientific, Cat # MA1-20832), anti-Ndc80 (rabbit polyclonal, 1:500, Life technologies, Cat # PA5-78319).

Validation

Validations of all primary antibodies for the species and application were provided in statements on the manufacturer's websites, as listed here:
https://www.sigmaaldrich.com/IT/it/product/mm/05623af488?gclid=Cj0KCQjwxYOiBhC9ARIsANiEIfZO9xJW57UBZCX73oZnKzXV6k7I8jtPI4w5M-k7TRidKzb-rO3FC08aAhP-EALw_wcB&gclsrc=aw.ds
chrome-extension://efaidnbmnnnibpcajpcglclefindmkaj/https://www.takarabio.com/documents/Certificate%20of%20Analysis/632592/632592-632593-080720.pdf
https://www.citeab.com/antibodies/835474-sc-6666-sir2-yn-19
https://www.thermofisher.com/antibody/product/GFP-Antibody-Polyclonal/A-6455
https://www.thermofisher.com/antibody/product/MA1-81357.html?gclid=Cj0KCQjwxYOiBhC9ARIsANiEIfZqlv2GUCbN_u6xxtRECGH9w9_7M7GZ4kmMABQG3K9IPcaApXVv5-IaAoRCEALw_wcB&ef_id=Cj0KCQjwxYOiBhC9ARIsANiEIfZqlv2GUCbN_u6xxtRECGH9w9_7M7GZ4kmMABQG3K9IPcaApXVv5-IaAoRCEALw_wcB:G:s&s_kwcid=AL!3652!3!459736943987!!!g!!!10950825775!106531320406&cid=bid_pca_aup_r01_co_cp1359_pjt0000_bid00000_0se_gaw_dy_pur_con
https://www.jacksonimmuno.com/catalog/products/715-165-151
https://www.jacksonimmuno.com/catalog/products/111-095-144
https://www.abcam.com/products/primary-antibodies/aurora-b-antibody-ab2254.html
https://www.citeab.com/antibodies/722546-ab13939-anti-cenpa-antibody-3-19-chip-grade
https://www.antibodiesinc.com/products/anti-centromere-protein-antibody-15-234
https://www.thermofisher.com/antibody/product/HEC1-Antibody-Polyclonal/PA5-78319
https://www.abnova.com/products/products_detail.asp?catalog_id=H00083732-B02P
https://www.sigmaaldrich.com/IT/it/product/sigma/t5168
https://www.thermofisher.com/antibody/product/Goat-anti-Mouse-IgG-H-L-Highly-Cross-Adsorbed-Secondary-Antibody-Polyclonal/A-21236
https://www.thermofisher.com/antibody/product/Goat-anti-Mouse-IgG-H-L-Cross-Adsorbed-Secondary-Antibody-Polyclonal/A-21425
https://www.thermofisher.com/antibody/product/Goat-anti-Rabbit-IgG-H-L-Highly-Cross-Adsorbed-Secondary-Antibody-Polyclonal/A-21245
https://www.citeab.com/antibodies/2400609-a-11013-goat-anti-human-igg-h-l-cross-adsorbed-sec
https://www.cosmobiousa.com/products/anti-aid-tag-iaa17-protein-mab
https://www.thermofisher.com/antibody/product/PGK1-Antibody-clone-22C5D8-Monoclonal/459250
https://www.biolegend.com/en-us/products/purified-anti-ha-11-epitope-tag-antibody-11374?GroupID=GROUP26
https://www.sigmaaldrich.com/IT/it/product/sigma/p1291
https://www.citeab.com/antibodies/3288338-p0161-rabbit-anti-mouse-immunoglobulins-hrp-ig-frac
https://www.cellsignal.com/products/primary-antibodies/gapdh-14c10-rabbit-mab/2118?gclid=Cj0KCQjwxYOiBhC9ARIsANiEIfbtjqp15V4JhTWhrexU5wYdpD6uWKYVomKAbVO2BM-qz9ACJScrzIoaAqV_EALw_wcB&gclsrc=aw.ds
https://www.citeab.com/antibodies/3288347-p0448-goat-anti-rabbit-immunoglobulins-hrp-affinity

Eukaryotic cell lines

Policy information about cell lines

Cell line source(s)

The human hTERT-immortalized RPE-1 cell line (ATCC, Cat # CRL-4000) used in this study was obtained from the RIKEN Cell Line Bank (Japan).

Authentication

The RIKEN Cell Line Bank (Japan) authenticated the cell line by short tandem repeat polymorphism analysis and provided an identity-validation report with the shipped cells.

Mycoplasma contamination

The cell line tested negative for mycoplasma contimination as performed by the cell culture facility at our department.

Commonly misidentified lines
(See ICLAC register)

Not applicable.

Flow Cytometry

Plots

Confirm that:

☒ The axis labels state the marker and fluorochrome used (e.g. CD4-FITC).

☒ The axis scales are clearly visible. Include numbers along axes only for bottom left plot of group (a 'group' is an analysis of identical markers).

☒ All plots are contour plots with outliers or pseudocolor plots.

☒ A numerical value for number of cells or percentage (with statistics) is provided.

Methodology

Sample preparation	The RPE-1 cells were harvested and centrifuged twice for 7 min at 1,000xgat room temperature. The cell pellet was re-suspended in 300µL 1xPBS and then fixed in 700µL pre-cooled ethanol 100%, added dropwise while gently vortexing. The samples were stored at -20°C until staining thier DNA with propidium iodide. Before staining, the cells were washed twice with 1xPBS, by centrifugation at 2000xg for 3 min at room temperature, each time aspirating and vortexing the pellet. The pellet was re-suspended in 250µL staining solution containing 10 µg/mL propidium iodide (Invitrogen, Cat. nr P3566-1.0 mg/mL (100x)) and 100 µg/mL PureLink™ RNase A (Invitrogen™, cat. nr. 12091021-20 mg/mL (200X)) diluted in 1xPBS. Samples were incubated for 30 min at 37°C and then stored at 4°C. Sample preparation is also described in the Methods section of our manuscript.
Instrument	FACS Calibur and FACS Aria III flow cytometers (BD Biosciences). Indicated also in the Methods section in our manuscript.
Software	The FACS data were analysed with ModFit LT 4.0 software (Verity Software House). Indicated also in the Methods section in our manuscript.
Cell population abundance	10,000-30,000 events were analysed for each sample.
Gating strategy	For data acquisition, we used forward and side scatter plots to identify the cells. Pulse shape analysis was used to identify clumps and doublets (this can be pulse area vs. pulse width or pulse area vs. pulse height depending on the cytometer used). For data analysis, gates were placed on the single cell populations using pulse width vs. pulse area to exclude doublets and debris (R1 in Supplementary Fig. 8c). This gate was then applied to the PI histogram plot (cell number vs PE-A or PE-H).

☒ Tick this box to confirm that a figure exemplifying the gating strategy is provided in the Supplementary Information.
